# Quality of care in prevention, detection and management of postpartum hemorrhage in hospitals in Afghanistan: an observational assessment

**DOI:** 10.1186/s12913-020-05342-y

**Published:** 2020-06-02

**Authors:** Nasratullah Ansari, Farzana Maruf, Partamin Manalai, Sheena Currie, Mohammad Samim Soroush, Sher Shah Amin, Ariel Higgins-Steele, Young Mi Kim, Jelle Stekelenburg, Jos van Roosmalen, Hannah Tappis

**Affiliations:** 1 Athena Institute, Faculty of Science, Vrije Universiteit, Amsterdam, the Netherlands; 2 Global Financing Facility, World Bank Group, Kabul, Afghanistan; 3 Jhpiego, Kabul, Afghanistan; 4 Jhpiego, 1615 Thames Street, Baltimore, MD USA; 5 Reproductive, Maternal, Newborn, Child and Adolescent Health Department, Ministry of Public Health, Masood Square, Kabul, Afghanistan; 6 USAID-Afghanistan, Masood Square, Kabul, Afghanistan; 7 UNICEF-Afghanistan, Jalalabad Road, Kabul, Afghanistan; 8 University Medical Centre Groningen, Department of Health Sciences, Global Health, University of Groningen, Groningen, the Netherlands; 9 Department of Obstetrics and Gynecology, Leeuwarden Medical Centre, Leeuwarden, the Netherlands

**Keywords:** Afghanistan, Postpartum hemorrhage, Quality of care, Maternal health, Emergency obstetric care

## Abstract

**Background:**

Hemorrhage is the leading cause of maternal mortality worldwide and accounts for 56% of maternal deaths in Afghanistan. Postpartum hemorrhage (PPH) is commonly caused by uterine atony, genital tract trauma, retained placenta, and coagulation disorders. The purpose of this study is to examine the quality of prevention, detection and management of PPH in both public and private hospitals in Afghanistan in 2016, and compare the quality of care in district hospitals with care in provincial, regional, and specialty hospitals.

**Methods:**

This study uses a subset of data from the 2016 Afghanistan National Maternal and Newborn Health Quality of Care Assessment. It covers a census of all accessible public hospitals, including 40 district hospitals, 27 provincial hospitals, five regional hospitals, and five specialty hospitals, as well as 10 purposively selected private hospitals.

**Results:**

All public and private hospitals reported 24 h/7 days a week service provision. Oxytocin was available in 90.0% of district hospitals, 89.2% of provincial, regional and specialty hospitals and all 10 private hospitals; misoprostol was available in 52.5% of district hospitals, 56.8% of provincial, regional and specialty hospitals and in all 10 private hospitals. For prevention of PPH, 73.3% women in district hospitals, 71.2% women at provincial, regional and specialty hospitals and 72.7% women at private hospital received uterotonics. Placenta and membranes were checked for completeness in almost half of women in all hospitals. Manual removal of placenta was performed in 97.8% women with retained placenta. Monitoring blood loss during the immediate postpartum period was performed in 48.4% of women in district hospitals, 36.9% of women in provincial, regional and specialty hospitals, and 43.3% in private hospitals. The most commonly observed cause of PPH was retained placenta followed by genital tract trauma and uterine atony.

**Conclusion:**

Gaps in performance of skilled birth attendants are substantial across public and private hospitals. Improving and retaining skills of health workers through on-site, continuous capacity development approaches and encouraging a culture of audit, learning and quality improvement may address clinical gaps and improve quality of PPH prevention, detection and management.

## Background

Obstetric hemorrhage is the leading cause of maternal mortality worldwide. It contributes to nearly one-third of all maternal deaths globally, the majority taking place in low-income countries [1]. An estimated 12% of postpartum hemorrhage (PPH) survivors will suffer from long- lasting, severe complications [2]. Contributing factors are many and often rooted in deficiencies in the health system, human resource capacity and supplies of commodities [3]. Homebirths without skilled birth attendants (SBAs), late transfer to health facilities with emergency obstetric care and the low-quality of care in these facilities are also contributing factors [4]. Furthermore, geographical, social and economic barriers limit women’s access to health services [5].

In Afghanistan, obstetric hemorrhage accounts for 56% of maternal deaths [6]. Afghanistan has one of the world’s highest maternal mortality ratios, estimated at 638 per 100,000 live births [7]. More than half (51%) of all births in Afghanistan take place at home without skilled attendants and only 43% of women give birth in public health facilities and 5% in private facilities [8]. Although Afghanistan has made substantial gains in the last 15 years in coverage of maternal health services and facility births as well as health system performance, further progress is still needed, especially with regard to the quality of basic and comprehensive emergency obstetric and newborn care [9, 10].

National health policies have prioritized quality improvement of maternal health care with respect to the major causes of maternal deaths, such as PPH [11]. National clinical guidelines to prevent, detect and manage PPH, based on the World Health Organization’s (WHO) recommendations, are available in the country; however, data on compliance with these national guidelines are scarce. WHO’s indicators for quality of prevention and management of PPH, such as uterotonics utilization, are not included in Afghanistan’s health information system [12]. Although an emergency obstetric and newborn care study in 2010 showed that the majority of hospitals had essential supplies and equipment and 85% of SBAs had received training on prevention of PPH before 2010, the present status of hospital readiness and SBA practices for prevention, detection and management of PPH in hospitals is unknown [13].

The purpose of this study is to examine the quality of prevention, detection and management of PPH in both public and private hospitals in 2016, and compare the quality of care in district hospitals with care in provincial, regional, and specialty hospitals.

## Methods

### Study design

The 2016 Afghanistan National Maternal and Newborn Health Quality of Care Assessment is a cross-sectional national survey. The assessment was designed to examine health facility readiness for basic and comprehensive emergency maternal and newborn care, and to assess quality of routine antenatal care (ANC), childbirth and postpartum care as well as management of selected obstetric and newborn complications. The focus of this study is on prevention, detection and management of PPH in public and private hospitals.

Five data collection tools (see online supplementary materials) were used: 1) facility inventory and record reviews to verify availability of medications, supplies, human resources, infrastructure and recordkeeping; 2) interviews to collect information on SBAs’ practices and constraints faced in the provision of labor and postnatal care; 3) labor and delivery observation checklists; 4) postnatal care observation checklists; and 5) PPH case management observation checklists.

Observation checklists were based on WHO guidelines and adapted from tools used in conducting quality of care assessments in other countries [14], in Demographic and Health Survey service provision assessments [15], and in emergency obstetric and newborn care assessments, supported by the Averting Maternal Death and Disability program [16]. The tools were developed in English and translated to local languages, Dari and Pashto.

### Sample

The study includes a census of all accessible public hospitals with an average of five or more births per day reported in the national health management information system for 2015: 40 district hospitals, 27 provincial hospitals, five regional hospitals, and five specialty hospitals. District hospitals provide primary healthcare and general medical and surgical services closer to the community. They are typically staffed by midwives, nurses, anesthesiologists, junior medical officers and surgeons. Provincial hospitals provide more sophisticated services for diagnosing and treating various conditions. Provincial hospitals support the use of some specialist doctors in the capital of the provinces. In addition to above, regional and specialty hospitals are tertiary hospitals that provide more advanced specialized care. Regional hospitals are located in five different regions. Specialty hospitals are mostly located in capital Kabul [17]. According to the Ministry of Public Health (MoPH), all hospitals should provide comprehensive emergency obstetric and neonatal care for 24 h per day and 7 days per week [11, 17]. Two additional district hospitals reported an average of five or more births per day in 2015 but were not accessible due to insecurity at the time of data collection. Ten private hospitals with an average of at least five births per day (two in each of Afghanistan’s five most densely populated provinces) were purposively sampled to provide a snapshot of services in the private sector.

### Data collection, sites and procedures

Data collectors were 32 experienced female doctors and midwives who received technical updates on maternal and newborn health care and training on data collection techniques with a focus on clinical observation, data quality assurance, research ethics and CommCare software (Dimagi, Cambridge, MA, USA).

Data collection was completed in a 2–3-day visit to each hospital between May and August 2016. Each hospital in-charge was informed about the purpose of the assessment and the data collection process by the data collectors upon arrival. In each district hospital teams of three data collectors interviewed up to five SBAs on day duty. In each provincial, regional and specialty hospital data collectors interviewed up to five SBAs on day duty and five SBAs on night duty. Data collectors observed up to five vaginal births in each district hospital and up to 10 in each provincial, regional and specialty hospital (five during a day shift and five during a night shift). Procedures from initial admission and client observation through the first hour postpartum were documented in labor and delivery checklists. Women’ examinations during postpartum ward rounds before discharge were documented in postpartum observation checklists. PPH cases attended during facility assessment visits were observed, including women who may have given birth at home.

Data collection was conducted using CommCare software loaded on Android tablets, with paper tools used as backup in sites where use of tablets was considered a security risk or unacceptable to care providers or women. Where paper tools were used, data from completed checklists were entered into the software when data collectors were in a safe location with internet access. Logic, skip and consistency checks were built into the program, and data collectors were trained to review records for missing or inconsistent answers before submission.

### Data analysis

Descriptive statistics were used to analyze indicators of interest and Ӽ^2^ tests were used for differences in hospitals’ readiness and SBAs’ performance on prevention and detection of PPH by hospital type. Stata® software version 15 was used for all statistical analysis with a type I error of 0.05. Data from private hospitals are based on purposively nonrandom sampling and not compared with the public health facilities.

Observations of PPH case management data were analyzed based on the causes of PPH and type of interventions. Interventions included administration of uterotonics, uterine massage, intravenous fluids, blood transfusion, additional procedures and case recording in clinical logbooks.

## Results

In district hospitals, 233 health providers were interviewed, in provincial, regional and specialty hospitals 315, and in private hospitals 48. The number of observations during the third stage of labor totaled 270 in district hospitals, 379 in provincial, regional and specialty hospitals, and 33 in private hospitals. In the inpatient postnatal ward, the number of observations for detection of PPH was 188 in district hospitals, 214 in provincial, regional and specialty hospitals, and 30 in private hospitals. PPH management was observed in 72 women in various inpatient settings.

### Characteristics of hospitals and health care providers

In all public and private hospitals, management reported provision of services 24 h a day, 7 days per week. The median number of births per month was 232 (142–1233) in district hospitals, 558 (76–2157) in provincial, regional and specialty hospitals, and 91 (8–218) in private hospitals. There was no statistically significant difference in caseload between the two types of public hospitals.

Providing uterotonics for management of PPH in the last 3 months was reported in 39 of 40 (97.5%) in district hospitals, 35 of 37 (94.6%) in provincial, regional and specialty hospitals, and 8 of 10 private hospitals. Meanwhile, 30 (75.0%) district hospitals, 36 (97.3%) provincial, regional and specialty hospitals, and seven private hospitals provided blood transfusion for maternity care. Provision of blood transfusion was higher in provincial, regional and specialty hospitals than in district hospitals (*p* = 0.019) (Table 1).

**Table 1:** Characteristics of public and private hospitals included in assessment

**Characteristics of Hospitals**	**Facility Type**			
	**District Hospitals*n*** **= 40**	**Provincial, Regional & Specialty Hospitals** ***n*** **= 37**	***p*-value**	**Private Hospitals*n*** **= 10**
Number (%) of hospitals providing uterotonic to manage PPH cases in the past 3 months	39 (97.5%)	35 (94.6%)	0.577	8 (80.0%)
Number (%) of hospitals having performed blood transfusion for maternity care in the past 3 months	30 (75.0%)	36 (97.3%)	**0.019**	7 (70.0%)
**Characteristics of health care providers**	**District Hospitals (*n*** **= 233 SBA)**	**Provincial, Regional, & Specialty Hospitals** **(*n*** **= 315 SBA)**	***p*-value**	**Private Hospitals (*n*** **= 48 SBA)**
Number (%) of SBAs having received training on basic emergency obstetric and newborn care in the past 3 years	43 (18.5%)	88 (27.9%)	**0.044**	16 (33.3%)
Number (%) of SBAs having received training on use of misoprostol for prevention and/or management of postpartum hemorrhage in the past 3 years	26 (11.2%)	54 (17.1%)	0.223	11 (22.9%)

In district hospitals, 43 of 233(18.5%) SBAs, in provincial, regional and specialty hospitals 88 of 315 (27.9%) SBAs and in private hospitals 16 of 48 (33.3%) SBAs received emergency obstetric and neonatal care (EmONC) training in the past 3 years. The number of SBAs trained on EmONC was higher in provincial, regional and specialty hospitals than in district hospitals (*p* = 0.044) (Table 1).

### Availability of medicines and guidelines for prevention and management of PPH

Oxytocin was available in 36 of 40 (90.0%) district hospitals, in 33 of 37 (89.2%) provincial, regional and specialty hospitals and all ten private hospitals. Misoprostol was available in 21 of 40 (52.5%) district hospitals, in 21 of 37 (56.8%) provincial, regional and specialty hospitals, and in all ten private hospitals (Table 2).

**Table 2:** Availability of guidelines and medicines for prevention and management of PPH at the point of care

Items available in the delivery room (%)	Facility Type			
	District Hospitals ***n*** = 40	Provincial, Regional & Specialty Hospitals ***n*** = 37	***p***-value	Private Hospitals, ***n*** = 10
Guidelines for emergency obstetric and newborn care	19 (47.5%)	16 (43.2%)	0.369	5 (50.0%)
Intravenous solutions: Ringers lactate, D5%NS, or NS infusion	35 (87.5%)	34 (91.9%)	0.376	9 (90.0%)
Injectable oxytocin	36 (90.0%)	33 (89.2%)	0.261	10 (100.0%)
Misoprostol	21 (52.5%)	21 (56.8%)	0.799	10 (100.0%)
Injectable ergometrine/methergine	24 (60.0%)	24 (64.9%)	0.457	8 (80.0%)

Less than half of the public hospitals (19 of 40 [47.5%] district hospitals, 16 of 37 [43.2%] provincial, regional and specialty hospitals) and 5 of 10 private hospitals had guidelines for EmONC (Table 2).

### Observation of prevention and detection practices of PPH during childbirth and immediate postpartum care

Among the births observed, 198 of 270 (73.3%) women in district hospitals, 270 of 379 (71.2%) women in provincial, regional and specialty hospitals and 24 of 33 (72.7%) women in private hospitals received uterotonics for prevention of PPH during the third stage of labor. Oxytocin was the most frequently used uterotonic in all hospitals. In 119 of 270 (44.1%) district hospitals, in 184 of 379 (48.6%) provincial, regional and specialty hospitals and in 16 of 33 (48.5%) private hospitals women received uterotonics within 1 min after birth (Table 3).

**Table 3:** SBA performance observed for prevention and detection of PPH in hospital

**PREVENTION PRACTICES**	**District Hospitals**	**Provincial, Regional & Specialty Hospitals**	***p*-value**	**Private Hospitals**
**Number (%) of observations during third stage of labor**	***n*** **= 270**	***n*** **= 379**		***n*** **= 33**
Uterotonic administered	198 (73.3%)	270 (71.2%)	0.211	24 (72.7%)
Oxytocin	188 (69.6%)	264 (70.0%)	0.297	21 (63.6%)
Misoprostol	2 (0.7%)	0 (0.0%)		1 (3.0%)
Ergometrine	0 (0.0%)	1 (0.3%)		0 (0.0%)
Recorded type not observed	8 (4.0%)	5 (1.4%)		2 (8.3%)
Uterotonic administered within one minute	119 (44.1%)	184 (48.6%)	0.121	16 (48.5%)
**DETECTION PRACTICES**	**District Hospitals**	**Provincial, Regional & Specialty Hospitals**	***p*-value**	**Private Hospitals (*n*** **= 38)**
**Number (%) of observation during third stage of labor and immediately postpartum**	***n*** **= 270**	***n*** **= 379**		***n*** **= 33**
Uterus checked immediately following the delivery of the placenta	203 (75.2%)	266 (70.2%)	0.369	23 (69.7%)
Placenta and membranes checked for completeness	135 (50.0%)	207 (54.6%)	0.524	17 (51.5%)
Perineum and vagina checked for tears	185 (68.5%)	285 (75.2%)	**0.039**	25 (75.8%)
Vital signs checked within 15 min	92 (34.1%)	102 (27.5%)	0.115	11 (33.4%)
Uterus palpated within 15 min	135 (50.0%)	165 (47.4%)	0.424	13 (37.1%)
**Number (%) of observations in inpatient postnatal ward**	***n*** **= 188**	***n*** **= 214**	***p*-value**	***n*** **= 30**
Client examined for excessive vaginal bleeding	91 (48.4%)	79 (36.9%)	**0.019**	13 (43.3%)
Client examination includes				
Taking pulse	81 (43.1%)	58 (27.1%)	**0.002**	16 (53.3%)
Taking blood pressure	141 (75.0%)	149 (69.6%)	0.065	23 (76.7%)
Checks fundus and massage if soft	129 (68.6%)	104 (48.6%)	**< 0.001**	21 (70.0%)

Uterine tone was checked within 15 min after birth in 135 of 270 (50.0%) women in district hospitals, 165 of 379 (47.4%) women in provincial, regional and specialty hospitals and 13 of 33 (37.1%) in private hospitals. Placenta and membranes were checked for completeness in 135 of 270 (50.0%) women in district hospitals, 207 of 379 (54.6%) in provincial, regional and specialty hospitals and 17 of 33 (51.5%) in private hospitals. More women in provincial, regional and specialty hospitals [285 of 379 (75.2%)] and in private hospitals (25 of 33; 75.8%) than in district hospitals (185 of 270; 68.5%) (*p* = 0.039) had their perineum and vagina checked for tears.

In the inpatient postnatal ward, the proportion of women examined for excessive bleeding during postpartum ward rounds in district hospitals (91 of 188; 48.4%) was significantly higher than in provincial, regional and specialty hospitals (79 of 214; 36.9%) (*p* = 0.019]. In private hospitals this proportion was 43.3% (13 of 30) women (Table 3).

### Observation of management of PPH in hospitals

A total of 72 women with hemorrhage were observed. One woman had antepartum hemorrhage and in three women, insufficient data were available to classify their conditions. Those four women were excluded from the analysis.

Of 68 women with PPH, 14 had homebirths and seven of them had hemorrhagic shock on admission. Two women gave birth on the way to hospital and one of them arrived in hemorrhagic shock. Fifty-two women gave birth in the hospitals and 11 of them developed hemorrhagic shock. No maternal death occurred. Files of 46 of 68 women could be traced in the clinical logbooks.

Active management of the third stage of labor (AMTSL) had been observed in 37 of 52 (71.2%) women who gave birth in hospital; 35 received 10 International Units (IU) of oxytocin, one received 15 IU oxytocin, and one 800 μg misoprostol. Fifteen of 52 (28.8%) women were not observed during labor and delivery, or did not receive AMTSL.

The most commonly observed cause of PPH was retained placenta (46 of 68; 67.6%) followed by genital tract trauma (12/68; 17.6%) and uterine atony (10/68; 14.7%). Eleven women with retained placenta also had genital tract injury (Table 4).

**Table 4:** Observation of Management of PPH in Hospitals

	Causes of PPH			Total
Type of Intervention	Uterine atony ***n*** = 10 (%)	Genital Tract tears ***n*** = 12 (%)	Retained placenta^a^ ***n*** = 46 (%)	***n*** = 68 (%)
**Uterotonics (excluding AMSTL)**	9 (90%)	6 (50%)	34 (74%)	49 (72%)
Oxytocin	7 (70%)	2 (17%)	25 (54%)	34 (50%)
Less than 40 IU	3 (30%)	1 (8%)	17 (37%)	21 (31%)
40 IU or more	4 (40%)	1 (8%)	4 (9%)	9 (13%)
not documented	–	–	4 (9%)	4 (6%)
Misoprostol	2 (20%)	4 (33%)	9 (20%)	15 (22%)
Less than 800 μg	–	2 (17%)	4 (9%)	6 (9%)
800 μg or more	1 (10%)	1 (8%)	3 (7%)	5 (7%)
not documented	1	1	2	4
**Uterine massage**				
Yes	10 (100%)	10 (83%)	40 (87%)	60 (88%)
No	–	2 (17%)	5 (11%)	7 (10%)
not documented	–	–	1	1
**IV fluids**				
Yes	9 (90%)	10 (83%)	42 (91%)	61 (90%)
No	1 (10%)	2 (17)	4 (9%)	7 (10%)
**Blood transfusion**				
Yes	2 (20%)	2 (17%)	14 (30%)	18 (26%)
1 unit or less	2 (20%)	2 (17%)	8 (17%)	12 (18%)
2 units or more	–	–	3 (7%)	3 (4%)
unknown	–	–	3	3
**Additional Procedure**				
Bimanual compression	3 (30%)	1 (8%)	5 (11%)	9 (13%)
Aortic compression	3 (30%)	–	6 (13%)	9 (13%)
Genital tract tears repair	N/A	10 (83%)	6 (13%)	16 (24%)
Removal of retained placenta/products	N/A	N/A	45 (98%)	45 (66%)
Tranexamic acid	2 (20%)	1 (8%)	1 (2%)	4 (6%)
Hysterectomy	–	1 (8%)	1 (2%)	2 (3%)
**Recorded as PPH case in the logbook**				
Yes	9 (90%)	7 (58%)	30 (65%)	46 (68%)
No	1 (10%)	5 (42%)	16 (35%)	22 (32%)

Note: all women’s outcome = alive

^a^Eleven women with retained placenta also had a perineal laceration

For management of PPH, uterotonics were administered in 9 of 10 women with uterine atony and 34 of 46 (74%) women with retained placenta cases. Oxytocin was given to 7 of 10 and misoprostol was given to 2 of 10 women with uterine atony. For management of retained placenta, oxytocin was administered in 25 of 46 (54%) and misoprostol 9 of 46 (20%) women. However, the oxytocin doses varied widely from less than 40 IU to 40 IU or more and misoprostol from less than 800 μg to 800 μm or more. Uterine massage was performed in all women with uterine atony and 40 of 46 (87%) women with retained placenta.

All women who were in shock (19 of 68; 28%) received IV fluids and 12 of 19 (63.1%) women received a blood transfusion.

Bimanual compression of the uterus (9 of 68; 13.2%) and aortic compression (9 of 68; 13.2%) were observed for management of PPH. Genital tract tears were repaired in 16 of 23 (69.5%) women.

Hysterectomy was performed in two cases. One woman was diagnosed with placenta accreta and subtotal hysterectomy was performed; she received two and half units of blood and 4000 ml IV fluids.

## Discussion

Numerous gaps in practices of SBAs with respect to prevention, detection and management of PPH were found in all hospitals. Although most facilities had oxytocin, various health facilities faced shortages of other supplies and medicines required for prevention and management of PPH.

Oxytocin is a drug of choice in prevention of PPH during third stage of labor and plays a central role in management of PPH [18]. Oxytocin was available in the majority of public and all private hospitals; however, a few public hospitals faced potentially serious shortages of oxytocin. Challenges in the supply chain in those hospitals could be the main reason. Misoprostol is included as a “special drug” for prevention of PPH in the MoPH Essential Drug List [19], but it is tightly controlled. Approximately half of all different hospitals had misoprostol in the labor room and there were no statistically significant differences between these hospital types. Surprisingly, all private hospitals had misoprostol in the labor room. It may be caused by private sector’s independent procurement mechanisms. Despite clear recommendations in the MoPH Reproductive, Maternal, Newborn Child and Adolescent Health Strategy 2017–2021 [11], public facilities still rely on a supply list issued in Essential Package for Hospitals Services issued in 2005, which does not include misoprostol [17]. WHO recommends use of misoprostol for prevention and management of PPH if oxytocin is unavailable or the bleeding does not respond to oxytocin [18]. It is critical for MoPH policy makers to review and improve the oxytocin supply chain, shift misoprostol from the special drug list to the essential drug list, and make both oxytocin and misoprostol available to address the shortage of commodities in all hospitals.

All women should receive uterotonics, preferably oxytocin as a component of AMTSL for prevention of PPH [18]. In our study, more than half (51%) women did not receive uterotonics within 1 min after birth as per WHO’s recommendations [18]. In a study covering six countries in sub-Saharan Africa, almost all women received oxytocin; however, similar to our study, only 52% received oxytocin within 1 min after birth [14]. Unfortunately, uterotonic administration for prevention of PPH in both public and private hospitals is relatively low in Afghanistan, leaving women at risk of PPH even within health facilities.

Early detection of PPH as per standard guidelines include checking placenta for completeness, uterine tone, excessive bleeding and genital tract tears [20]. Often SBAs did not check the placenta for completeness, monitor the uterine tone and vital signs after birth, or examine women for excessive bleeding during the immediate postpartum period in all public and private hospitals. Checking for genital tract tears was not performed in about one-quarter of births observed in public hospitals; provincial, regional and specialty hospitals, however, were performing slightly better than district hospitals. In a study in Madagascar, similar gaps were found in performance of health providers [21]. Early detection of PPH requires skilled care and rigorous monitoring; maternal monitoring appears to be weak in hospitals in Afghanistan, endangering women’s lives. Supportive strategies are needed to ensure SBAs take time to include evidence-based practices for detection of obstetric complications in routine care for every woman.

In observations of PPH management, we found that retained placenta was the most common cause of PPH; however, generally, uterine atony is the most frequent cause [22]. Most likely, our finding is explained by underreporting of PPH due to atony since blood loss is not measured regularly.

SBA performance during PPH management was inadequate. Uterotonics were not used in all PPH cases. Uterotonic dosage varied and did not adhere to national guidelines. Gaps in PPH management practices of SBAs are also common in other low-resource settings. For example in Kenya, health care providers missed repair of genital tract tears in a number of PPH cases [23]. In Tanzania, providers correctly followed the initial steps of PPH management, such as uterotonic administration, uterine massage, and initiation of IV fluid. They were, however, less consistent in adherence to other standard protocols for management of PPH [24].

A number of factors affect the performance of SBAs in provision of quality of care. These include lack of training opportunities, increased workload, poor supervision, low salaries, poor living conditions and lack of equipment and supplies [25].

Gaps in practices of SBAs in our study could be caused by lack of capacity building opportunities and a weak system to ensure provider competencies. We found that access to training to improve knowledge and skills in EmONC was limited for most SBAs, and fewer SBAs in district hospitals received training as compared to SBAs in provincial, regional and specialty hospitals in the past 3 years. This was in contrast to 2010, when most SBAs had recently received training on EmONC, including AMTSL and management of PPH in all types of public facilities [13].. Inadequacy of training could be related to lack of financial resources in Afghanistan. The national health accounts studies in 2011 and 2014 showed that expenditure on education and training for health providers was more than 50% lower in 2014 compared to 2011 [26, 27]. Considering scarcity of financial resources, innovative and cost-effective capacity development approaches should be reviewed and adopted. For instance, a recent study in Ghana indicates that low-dose, high-frequency training through on-site learning and practice, supported by mentors, decreases newborn mortality and intra-partum stillbirths and retains the knowledge and skills of health providers for longer than a year [28]. The low-dose, high-frequency training approach has proven to be cost effective [29]. In addition, evidence suggests that obstetric simulation-based training can reduce the incidence of PPH and improve the performance of providers in PPH management [30].

Policy makers and public health managers should review the existing MoPH human resource development strategy and adopt recent evidence-based, cost-effective and on-site capacity building approaches to effectively transfer learning and retain knowledge and skills of SBAs. Attention to documentation of clinical care and increased accountability for quality of care at all levels of the health system can also drive quality improvement efforts.

This assessment has several methodological limitations. Although the study sample was national in scope, it was not designed to provide a representative sample of private hospitals. Therefore, readiness and quality of care in public hospitals cannot be compared with private hospitals. In addition, findings can only be generalized to district hospitals with at least five births per day, not all district hospitals. We cannot link specific providers to case observations in the study dataset—interviews provide a picture of availability of guidelines and medicines in hospitals, observations provide a picture of observed care. Although efforts were made to ensure multiple providers were observed over a course of 2 days, the same provider may have been observed with multiple clients. There may be influence of the Hawthorne effect in observations of provider performance [31]. The study may therefore give an underestimation of gaps in providing inappropriate care. Despite these limitations, this is the first country-wide assessment that included direct observation of prevention, detection and management of PPH in hospitals in Afghanistan. Moreover, it is the first study of any kind to assess quality of PPH prevention, detection and management in private facilities in Afghanistan.

## Conclusions

Numerous gaps in quality of care for prevention, detection and management of PPH exist in all hospitals with limited differences between district hospitals and provincial, regional and specialty hospitals in Afghanistan.

Progress towards national and global development goals for improvement of maternal health will require dedicated efforts to improve prevention, detection and management of PPH in Afghanistan. Improving quality of care should be top priority for policy makers, health managers and service providers to reduce the high risks of maternal mortality and morbidity and meet the health needs of women. Improving and retaining skills of SBAs through on-site, continuous capacity development approaches and encouraging a culture of audit, learning and quality improvement may address clinical gaps and improve quality of PPH prevention, detection and management.

## Supplementary information

**Additional file 1.**

## Data Availability

Data is available from the Ministry of Public Health upon request. Requests should be directed to the MoPH Evaluation and Health Information Systems Department (ehis.moph@gmail.com).

## Abbreviations

AMTSL: Active management of the third stage of labor
ANC: Antenatal care
EmONC: Emergency obstetric and newborn care
IU: International unit
MoPH: Ministry of Public Health
PPH: Postpartum hemorrhage
SBA: Skilled birth attendant
WHO: World Health Organization
