# Supplementary material for: Quality of care in prevention, detection and management of postpartum hemorrhage in hospitals in Afghanistan: an observational assessment
Source: BMC Health Serv Res. 2020 Jun 2;20:484. doi: 10.1186/s12913-020-05342-y (PMC7265625; doi:10.1186/s12913-020-05342-y)
Supplement: Supplementary file 1 — Additional file 1. [file 12913_2020_5342_MOESM1_ESM.pdf]

2016 Afghanistan National Maternal and Newborn Health Quality of Care Assessment

DATA COLLECTION TOOLS

TOOL A: **Facility Inventory and Record Review**

TOOL B: **Skilled Birth Attendant Interview and Knowledge Test**

TOOL C: **Antenatal Care Observation Checklist**

TOOL D: **Labor and Delivery Care Observation Checklist**

TOOL D1: **Severe Pre-Eclampsia and Eclampsia Case Management Observation Checklist**

TOOL D2: **Postpartum Hemorrhage Case Management Observation Checklist**

TOOL D3: **Newborn Resuscitation Observation Checklist**

TOOL E: **Pre-discharge Postnatal Care Observation Checklist**

FOR ADDITIONAL INFORMATION, PLEASE CONTACT:
Dr. Hannah Tappis, Principal Investigator, Jhpiego (Hannah.Tappis@jhpiego.org)
Dr. Faridullah Atiqzai, HEMAYAT Deputy Chief of Party, Jhpiego (Faridullah.Atiqzai@jhpiego.org)

Health facility visited (name):						
Health facility code (from HMIS and/or facility listing):						

TYPE OF HEALTH FACILITY	
Specialized hospital .... [1]<br>Regional hospital ....[2]<br>Provincial hospital .... [3]<br>District hospital .... [4]<br>Comprehensive health center.................. [5]<br>Basic health center................................... [6]<br>Sub health center .... [7]	Private facility .... [8]<br>Family Health House...[9]<br>MCH or RH House..[10]<br>Birth Center... [11]<br>Other (specify) .... [12]<br>____
Province Name:	
District Name:	
City / Village Name:	
Name of Observer:	
Name of Team Leader	
Date of Visit: (dd/mm/yy)	__ __ / __ __ / __ __ __ __
Time of Visit: (hh:mm/am-pm)	__ __ :__ __ / __ __
Signature of Team Leader:	

EXPLAIN TO THE HEALTH WORKER THAT HIS/HER NAME WAS PROVIDED AS A KNOWLEDGEABLE MATERNAL AND/OR NEONATAL HEALTH PROVIDER AVAILABLE ON THAT DAY. VALIDATE WITH THE HEALTH WORKER THAT HE/SHE IS FAMILIAR WITH THE MATERNAL AND/OR NEONATAL HEALTH SERVICES IN THIS FACILITY.

Hello, I am _________________. I am representing the Ministry of Public Health and the USAID-funded HEMAYAT Project. We are conducting a study of health facilities in this country, with the goal of finding ways to improve maternal and newborn health services. We are asking for your cooperation and assistance to conduct an inventory of the supplies and equipment in place for antenatal care, labor and delivery care, and postnatal care at this facility. We will also examine the systems for referrals and record keeping. This should take no more than two hours.

There will be no direct benefit to you from assisting with this activity. Your name will not be recorded. The information collected may be used by the Ministry of Public Health or other organizations to improve services.

Do you have any questions for me? May we begin?

Section 1: General Facility Readiness

ASK QUESTIONS ALOUD TO FACILITY DIRECTOR/IN-CHARGE

Question	Yes	No	DK	Go to
A100: Is there a health care worker present at the facility or on call at all times?	1	0	98	
A101: Does this facility have a staffing plan (schedule) for staff providing 24 hour services?	**CODE**			
No	0			
Yes, present, schedule observed	1			
Yes, present, schedule reported, not seen	2			
Yes, on-call schedule observed	3			
Yes, on-call schedule reported, not seen	4			

READ ALOUD: PLEASE TELL ME HOW MANY STAFF IN EACH OF THE FOLLOWING OCCUPATIONAL CATEGORIES ARE CURRENTLY ASSIGNED TO, EMPLOYED BY, OR SECONDED TO THIS FACILITY, AND HOW MANY ARE ON DUTY AT GIVEN TIMES.

	a. Currently Assigned or Employed	b. On day duty S-Th	c. On night duty S-Th	d. On day duty Fri-Sat	e. On night duty Fri-Sat
A102: Generalist (non-specialist) medical doctor - male	__ __	__ __	__ __	__ __	__ __
A103: Generalist (non-specialist) medical doctor - female	__ __	__ __	__ __	__ __	__ __
A104: Obstetrician/ gynecologist	__ __	__ __	__ __	__ __	__ __
A105: General surgeon	__ __	__ __	__ __	__ __	__ __
A106: Pediatrician	__ __	__ __	__ __	__ __	__ __
A107: Other medical specialist	__ __	__ __	__ __	__ __	__ __
A108: Midwife or community midwife	__ __	__ __	__ __	__ __	__ __
A109: Anesthetist	__ __	__ __	__ __	__ __	__ __
A110: Laboratory scientist or technician (degree)	__ __	__ __	__ __	__ __	__ __

Question	Yes	No	DK	Go to
A111: Does the health facility have an easily visible Charter of Patients' Rights on display?	1	0	98	
A112: Does the health facility have a functional client complaint or feedback reporting mechanism?	1	0	98	
A113: Does the health facility have a functional employee complaint reporting mechanism?	1	0	98	

END OF SECTION 1

SECTION 2: Referral System Readiness

The next few questions I'd like to ask you are related to referral systems.

Question	Yes	No	DK	Go to
A200: Does this facility ever refer a women or newborn to another facility for care?	1	0	98	A200 is No → END SECTION
A201: When referring a patient to a higher level of care, how often does staff call ahead to inform the receiving facility that the patient is coming? *(READ ANSWER OPTIONS)*	**CODE**			
Never	0			If No→A203
Rarely	1			
Sometimes	2			
Usually	3			
Don't know	98			If DK→A203
A202: How does staff communicate with the referral clinic or hospital to alert them about the patient's arrival?	**CODE**			
Phone/radio	1			
Text message	2			
Don't know	98			
A203: To transport emergency patients from this facility, what strategies does this facility use? Does it… *(READ EACH ITEM)*				
A203a: Have its own means of transportation?	1	0	98	If No→A209
A203b: Have agreements with private taxis, cares, trucks or motorcycles?	1	0	98	
A203c: Request vehicles from the District Health Office?	1	0	98	
A203d: Request vehicles from the Provincial Health Office?	1	0	98	
A203e: Request vehicles from the local shura?	1	0	98	
A203f: Assume patient's family will arrange their own transport?	1	0	98	
A204: Does this facility have an ambulance on-site for emergency transportation of clients? *IF YES, ASK IF THE VEHICLE IS FUNCTIONING AND IF THERE IS FUEL AVAILABLE. (ACCEPT REPORTED RESPONSE.)*				
No	0			If No→A206
Yes	1			
Yes, not functioning or no fuel	2			
Yes, functioning with fuel but no driver on staff	3			
A205: Are there clear criteria for use of emergency transport? *(ASK FOR EXPLANATION OF CRITERIA AND TO SEE WRITTEN PROTOCOLS AND LOG BOOKS, IF AVAILABLE)*				
No	0			
Yes, criteria explained	1			
Yes, written protocols and/or log books observed	2			
A206: Does this facility have other transportation available on-site for emergency transportation of clients?				
No	0			If No→A209
Other motor vehicle	1			
Motorbike ambulance	2			
Bicycle ambulance	3			
Animal drawn cart	4			
Stretcher	5			
A207: Does the health facility have a driver on staff?	1	0	98	If No→A209
A208: How often is a driver available to transport a patient with an emergency? *(READ ANSWER OPTIONS)*				

Never	0			
Rarely	1			
Sometimes	2			
Usually	3			
Don't know	98			
A209: When patients are referred out to a higher level facility, how often do they leave accompanied with a referral form? *(READ ANSWER OPTIONS)*	**CODE**			
Never	0			If Never→A211
Rarely	1			
Sometimes	2			
Usually	3			
Don't know	98			If DK→A211
**Question**	**Yes**	**No**	**DK**	**Go to**
A210: Is the form standardized (used at other facilities too) or is it a form used only in this facility?	**CODE**			
Standardized	1			
Used only by this facility	2			
Both types are used	3			
Don't know	98			
A211: After this facility refers a patient, how often do you receive feedback about the treatment or outcomes of that patient? *(READ ANSWER OPTIONS)*	**CODE**			
Never	0			
Rarely	1			
Sometimes	2			
Usually	3			
Don't know	98			
A212: How often does a health worker accompany the patient being referred? *(READ ANSWER OPTIONS)*	**CODE**			
Never	0			If Never→A214
Rarely	1			
Sometimes	2			
Usually	3			
Don't know	98			If DK→A214
A213: What type of health worker accompanies the patients?				
A213a: Doctor	1	0	98	
A213b: Midwife	1	0	98	
A213c: Nurse	1	0	98	
A213d: Community health supervisor	1	0	98	
A213e: Community health worker	1	0	98	
A214: What is the MOST common maternal or fetal condition that requires referral out from this health facility?<br>A: Hemorrhage<br>B: Sepsis<br>C: Prolonged/obstructed labor<br>D: Pre-eclampsia/eclampsia<br>E: Premature rupture of membranes<br>F: Pre-term labor<br>G: Fetal distress<br>H: Previous caesarean surgery<br>I: Big baby<br>J: Other				
A214b: What is the SECOND MOST common maternal or fetal				

condition that requires referral out from this health facility?	
A: Hemorrhage	
B: Sepsis	
C: Prolonged/obstructed labor	
D: Pre-eclampsia/eclampsia	
E: Premature rupture of membranes	
F: Pre-term labor	
G: Fetal distress	
H: Previous caesarian surgery	
I: Big baby	
J: Other	
A215: What is the MOST COMMON postnatal newborn condition that requires referral out from this health facility?<br>A: Low birth weight<br>B: Prematurity<br>C: Respiratory difficulties<br>D: Sepsis<br>E: Jaundice<br>F: Asphyxia<br>G: Congenital abnormalities<br>J: Other	
A215b: What is the SECOND MOST common postnatal newborn condition that requires referral out from this health facility?	
A: Low birth weight	
B: Prematurity	
C: Respiratory difficulties	
D: Sepsis	
E: Jaundice	
F: Asphyxia	
G: Congenital abnormalities	
J: Other	
**END OF SECTION 2**	

SECTION 3: Labor and Delivery Service Readiness

ASK TO SPEAK WITH THE HEAD OF LABOR & DELIVERY UNIT (THIS MAY BE DIRECTOR/IN-CHARGE IF NO HEAD OF UNIT)

Question	Yes	No	DK	Go to
A300: Does this facility provide delivery services?	1	0		A300 is No → END SECTION
A301: Does this facility provide 24 hour coverage for delivery services?	1	0		No→A303
A302: Is a skilled birth attendant present at the facility or on call 24 hours a day, including weekends, to provide delivery care?	**CODE**			
No	0			
Yes, present, schedule observed	1			
Yes, present, schedule reported, not seen	2			
Yes, on-call schedule observed	3			
Yes, on-call, schedule reported, not seen	4			
A303: What is the usual mode of transport to bring in women in labor?	**CODE**			
Ambulance	1			
Own Motor Vehicle	2			
Rented Motor Vehicle	3			
Motorbike	4			
Animal drawn cart	5			
Other	95 (*Specify*):_______________________			
A304: Are there any fees/costs for women coming to this facility for delivery?	1	0	98	
A304a: Is there a formal payment required for labor and delivery services?	1	0	98	
A304b: Is a woman or her family expected to buy supplies for a normal delivery?	1	0	98	
A304c: In an obstetric/gynecological emergency, is a woman or her family expected to buy medicine or supplies prior to treatment?	1	0	98	
A305: How many hours do women generally stay at the facility following a normal delivery?	__ __ hours	(Enter 98 if don't know)		

READ ALOUD: NOW I AM GOING TO ASK YOU ABOUT MEDICAL INTERVENTIONS FOR MANAGEMENT OF COMPLICATIONS DURING LABOR AND DELIVERY. FOR EACH INTERVENTION, PLEASE TELL ME IF THIS IS EVER PROVIDED AT THIS FACILITY, AND IF YES, IF IT HAS BEEN CONDUCTED IN THIS FACILITY WITHIN THE PAST 3 MONTHS.

Question	Yes	No	DK	Go to
A306: Does this facility ever provide uterotonic to prevent postpartum hemorrhage?	1	0		No→A307
A306a: In the past 3 months?	1	0	98	No→A307
A306b: Which type of uterotonic?				
Oxytocin	1	0		
Ergometrine	1	0		
Misoprostol	1	0		
Other (specify)_________	1	0		
A307: Is misoprostol used in this facility for obstetric indications?	1	0		No→A308
A307a: For labor induction?	1	0	98	
A307b: For prevention of post-partum hemorrhage?	1	0	98	
A307c: For treatment of post-partum hemorrhage?	1	0	98	
A307d: For treatment of incomplete abortion?	1	0	98	
A308: Do health workers at this facility ever provide parenteral anticonvulsants for pregnancy-related hypertension?	1	0		No→A309
A308a: In the past 3 months?	1	0	98	
A308b: Which type of anticonvulsant?	**CODE**			
Magnesium sulfate	1			
Diazepam	2			
Both	3			

Other	95	(specify)_________		
Question	Yes	No	DK	Go to
A309: Do health workers at this facility ever provide parenteral antibiotics for pregnancy-related infections?	1	0		No→A310
A309a: In the past 3 months?	1	0	98	
A310: Do health workers at this facility ever perform Manual removal of placenta?	1	0		No→A311
A310a: In the past 3 months?	1	0	98	
A311: Do health workers at this facility ever perform Manual Vacuum Aspiration (MVA)?	1	0		No→A312
A311a: In the past 3 months?	1	0	98	
A312: Do health workers at this facility ever perform dilation and curettage (D&C) or evacuation and curettage (E&C)?	1	0		No→A313
A312a: In the past 3 months?	1	0	98	
A313: Do health workers at this facility use partographs to manage labor?	1	0		No→A314
A313a: In the past 3 months?	1	0	98	
A314: Do health workers at this facility ever perform assisted deliveries—that is, use forceps or vacuum extractor?	1	0		No→A315
A314a: In the past 3 months?	1	0	98	
A314b: Which type of instrument was most commonly used?	**CODE**			
Vacuum extractor	1			
Forceps	2			
Both	3			
A315: Do health workers at this facility ever perform caesarean sections?	1	0		No→A316
A315a: In the past 3 months?	1	0	98	
A315b: Is there a trained health worker who can perform a caesarean present in the facility or on call 24 hours a day (including weekends)?	**CODE**			
No	0			
Yes, present, schedule observed	1			
Yes, present, schedule reported, not seen	2			
Yes, on-call schedule observed	3			
Yes, on-call, schedule reported, not seen	4			
A315c: Is there an anesthetist present in the facility or on call 24 hours a day (including weekends)?	**CODE**			
No	0			
Yes, present, schedule observed	1			
Yes, present, schedule reported, not seen	2			
Yes, on-call schedule observed	3			
Yes, on-call, schedule reported, not seen	4			
A316: Do health workers at this health facility perform blood transfusions? (IF YES, is there a blood bank or are there transfusion services only)				
No blood transfusions	0			→A317
Yes, blood bank	1			
Yes, transfusion, no blood bank	2			
A316a: Has blood transfusion been performed for maternity care by this facility during the past 3 months?	1	0	98	
A317: Do health workers at this facility ever perform breech deliveries?	1	0		No→A318
A317a: In the past 3 months?	1	0	98	
A318: Do health workers at this facility ever perform episiotomies?	1	0		No→A319
A318a: In the past 3 months?	1	0	98	
A319: Do health workers at this facility ever perform simple fistula repairs?	1	0		No→A320
A319a: In the past 3 months?	1	0	98	
A320: Do health workers at this facility ever perform craniotomies?	1	0		No→A321
A320a: In the past 3 months?	1	0	98	

Question	Yes	No	DK	Go to
A321: Do health workers at this facility ever provide antenatal corticosteroids for fetal lung maturation?	1	0		No→A322
A321a: In the past 3 months?	1	0	98	
A322: Do health workers at this facility ever perform newborn resuscitation?	1	0		No→A323
A322a: In the past 3 months?	1	0	98	
A323: Do health workers at this facility ever support kangaroo mother care for pre-term and low birth-weight newborns?	1	0		No→A324
A323a: In the past 3 months?	1	0	98	
A324: Do health workers at this facility ever provide injectable antibiotics for treatment of severe newborn infection	1	0		No→A325
A324a: In the past 3 months?	1	0	98	
A325: Do health workers at this facility ever administer oxygen to a newborn?	1	0		No→A326
A325a: In the past 3 months?	1	0	98	
A326: Do health workers at this facility ever administer IV fluids to a newborn?	1	0		No→A327
A326a: In the past 3 months?	1	0	98	
A327: Do health workers at this facility ever support alternative feeding for newborns (expressing breast milk and using a cup or spoon for feeding)?	1	0		No→A328
A327a: In the past 3 months?	1	0	98	

READ ALOUD: NOW I WANT TO ASK YOU ABOUT HOW THIS FACILITY HANDLES CONTAMINATED REUSABLE EQUIPMENT AFTER COMPLETING A DELIVERY. IF THE UNIT PROCESSES SOME EQUIPMENT AND SENDS OTHER EQUIPMENT ELSEWHERE, INDICATE THE PROCEDURE FOR EQUIPMENT PROCESSED IN THIS SERVICE DELIVERY UNIT. IF VAGINAL DELIVERIES ARE CONDUCTED IN A DIFFERENT ROOM THAN CAESAREAN SECTION DELIVERIES, ASSESS THE PROCESSING EQUIPMENT FOR VAGINAL DELIVERIES.

A328: After completing a delivery, what procedures do health workers follow for initial handling of contaminated equipment (such as scissors, clamps) that will be reused?	**Code**
Nothing is done	0
Decontaminate in 0.5% chlorine solution, then soap & water scrub, then rinse	1
Soap & water scrub, then decontaminate	2
Soap & water brush scrub only	3
Disinfectant soak, not scrubbed	4
Soap & water, not brush scrubbed	5
Other (*specify*) ________________________________	95
Don't know	98

A329: Besides decontaminating and cleaning, what is the final process most commonly used for disinfecting or sterilizing medical equipment (such as surgical instruments) before they are reused? If different methods are used for different types of equipment, indicate the method(s) used for metal equipment such as speculums or forceps.	**Code**
Nothing is done	0
Dry-heat sterilization	1
Autoclaving	2
Steam sterilization	3
Boiling	4
Chemical method	5
Other (*specify*) ____________________________________	95
Don't know	98

THERE ARE NO MORE QUESTIONS FOR THE HEAD OF LABOR & DELIVERY UNIT/DIRECTOR. EXPLAIN THAT FOR THE NEXT SECTION, YOU WILL NEED TO WALK AROUND AND LOOK AT THE DELIVERY SERVICE AREA. THEY CAN NOW CHOOSE TO ACCOMPANY YOU FOR THE REST OF THE ASSESSMENT OR ATTEND TO OTHER BUSINESS. IF THEY DO NOT ACCOMPANY YOU, ASK IF A HEALTH WORKER INVOLVED IN DELIVERY CARE CAN HELP YOU WITH THE NEXT PART OF THE ASSESSMENT.

ASK TO SEE THE ROOM WHERE NORMAL DELIVERIES ARE CONDUCTED.

Question	Yes	No	DK	Go to
A330: Is there a place for hand washing in the labor room?	1	0		
A331: Is a newborn resuscitation table / newborn corner visible and easily accessible in the labor room?	1	0		
A332: Describe the area that sterilized equipment is stored in:<br>Cupboard with doors<br>Open shelves<br>Container with cover<br>Separate area well organized				
A333: Describe the setting of the delivery room	**Code**			
Private room with visual and auditory privacy	1			
Non-private room with visual and auditory privacy	2			
Visual privacy only	3			
No privacy	4			

NOTE THE AVAILABILITY AND CONDITION OF THE FOLLOWING SUPPLIES, EQUIPMENT AND MEDICATIONS NEEDED FOR DELIVERY SERVICES. ITEMS MAY BE IN DELIVERY ROOM OR AN ADJACENT ROOM. IF YOU DO NOT SEE AN ITEM, ASK THE HEALTH WORKER HELPING YOU TO SHOW YOU THE ITEM.

SUPPLIES AND EQUIPMENT IN DELIVERY ROOM	Observed	Reported not seen	Not available	Don't know	Go to
A334: Sterile gloves	1	2	3	98	
A335: Sharps container	1	2	3	98	
A336: Already mixed decontaminating solution (0.5% chlorine)	1	2	3	98	
A337: Alcohol hand rub	1	2	3	98	
A338: Waste receptacle with lid and plastic liner	1	2	3	98	
A339: Soap for hand washing	1	2	3	98	
A340: Water for hand washing	1	2	3	98	Not available/DK→A342
A341: How is water being made available for use in the delivery service area today?	**Code**				
Piped	1				
Bucket with tap	2				
Bucket or basin	3				
Don't Know	98				
A342: Syringes and needles	1	2	3	98	
A343: Sterile scissors or blade	1	2	3	98	
A344: Sterile disposable cord ties or clamps	1	2	3	98	
A345: Towel or blanket to wrap baby	1	2	3	98	
A346: Blank partographs or blank maternity booklets with partograph included	1	2	3	98	

Availability and Functioning of Supplies and Equipment	Availability				If Not seen, not available, DK	a. Functioning *		
	Observed	Reported not seen	Not available	Don't know		Yes	No	DK
A347: Incubator	1	2	3	98	Go to →A348	1	2	98
A348: Other source of heat for	1	2	3	98	Go to →A349	1	2	98

premature infant								
A349: Bag and mask (infant size) for resuscitation								
A349a: Bag	1	2	3	98	Go to →A349b	1	2	98
A349b: Mask size 0	1	2	3	98	Go to →A349c	1	2	98
A349c: Mask size 1	1	2	3	98	Go to →A350	1	2	98
	**Availability**					**a. Functioning ***		
**Availability and Functioning of Supplies and Equipment**	**Observed**	**Reported not seen**	**Not available**	**Don't know**	**If Not seen, not available, DK**	**Yes**	**No**	**DK**
A350: Suction device for mucus extraction	1	2	3	98	Go to →A351	1	2	98
A351: Suction apparatus for use with catheter (electric or manual)	1	2	3	98	Go to →A352	1	2	98
A352: Newborn resuscitation table with light source	1	2	3	98	Go to →A353	1	2	98
A353: Uninterrupted oxygen supply with regulator	1	2	3	98	Go to →A354	1	2	98
A354: Obstetric Forceps	1	2	3	98	Go to →A355	1	2	98
A355: Vacuum extractor (manual or electrical)	1	2	3	98	Go to →A356	1	2	98
A356: Manual vacuum aspirator (MVA) and cannula	1	2	3	98	Go to →A357	1	2	98
A357: Dilatation and curettage (D&C) kit	1	2	3	98	Go to →A358	1	2	98
A358: PPIUCD insertion kit	1	2	3	98	Go to →A359	1	2	98

IF MEDICATIONS ARE PACKAGED TOGETHER IN A COMBO-PACK FOR DELIVERIES, CIRCLE 1 FOR "OBSERVED >/ 1 VALID DOSE" FOR EACH INDIVIDUAL MEDICATION IN THE PACK

**MEDICATIONS IN DELIVERY ROOM**	**Observed ≥1 valid dose**	**Reported not seen**	**Not available**	**Don't know**
A359: Intravenous solutions: either Ringers lactate, D5NS, or NS infusion	1	2	3	98
A360: Injectable ergometrine/ methergine	1	2	3	98
A361: Injectable oxytocin	1	2	3	98
A362: Misoprostol	1	2	3	98
A363: Injectable diazepam	1	2	3	98
A364: Injectable magnesium sulfate	1	2	3	98
A365: Injectable Ca Gluconate	1	2	3	98
A366: Amoxicillin	1	2	3	98
A367: Injectable ampicillin	1	2	3	98
A368: Injectable gentamicin	1	2	3	98
A369: Lignocaine 1 or 2%	1	2	3	98
A370: Tetracycline ointment	1	2	3	98
A371: Dexamethasone/betamethasone	1	2	3	98
A372: Chlorhexidine gel	1	2	3	98
A373: Injectable vitamin K	1	2	3	98
**GUIDELINES/ PROTOCOLS IN DELIVERY ROOM**	**Observed**	**Reported not seen**	**Not available**	**Don't know**
A374: Guidelines or national treatment protocol for care/managing normal labor and birth (can be printed or handmade)	1	2	3	98

A375: Guidelines or national treatment protocol for essential newborn care (can be printed or handmade)	1	2	3	98
A376: Guidelines or national treatment protocol for emergency obstetric and newborn care (can be printed or handmade	1	2	3	98
A377: Guidelines for the pre-referral management of major obstetric and newborn complications (can be printed or handmade)	1	2	3	98

IF A315 = YES (HEALTH WORKERS AT THIS FACILITY PERFORM CESAREAN SURGERIES, ASK TO SEE THE ROOM WHERE SURGERIES ARE PERFORMED. IF THERE IS NO ROOM FOR SURGERIES, LOOK IN THE DELIVERY ROOM.
CHECK IF THE FOLLOWING EQUIPMENT, SUPPLIES AND MEDICATIONS ARE AVAILABLE IN THE ROOM OR IN AN ADJACENT ROOM.

EQUIPMENT IN SURGICAL SERVICE AREA	Availability				If Not seen, not available, DK	a. Functioning *		
	Observed	Reported not seen	Not available	Don't know		Yes	No	DK
A378: Operating Table	1	2	3	98	Go to→A379	1	2	98
A379: Operating light	1	2	3	98	Go to→A380	1	2	98
A380: Anesthesia giving set	1	2	3	98	Go to→A381	1	2	98
A381: Scrub area adjacent to or in the operating room	1	2	3	98				
A382: Tray, drum, or package with sterilized instruments ready for use	1	2	3	98				
A383: Bag and mask (infant size) for resuscitation								
A383a: Bag	1	2	3	98	Go to →A383b	1	2	98
A383b: Mask size 0	1	2	3	98	Go to →A383c	1	2	98
A383c: Mask size 1	1	2	3	98	Go to →A384	1	2	98
A384: Suction device for mucus extraction	1	2	3	98	Go to →A385	1	2	98
A385: Suction apparatus for use with catheter (electric or manual)	1	2	3	98	Go to →A386	1	2	98
A386: Newborn resuscitation table with light source	1	2	3	98	Go to →A387	1	2	98
A387: Uninterrupted oxygen supply with regulator	1	2	3	98	Go to →A388	1	2	98

MEDICATIONS IN SURGICAL SERVICE AREA	Observed ≥1 valid dose	Reported not seen	Not available	Don't know
A388: Halothane	1	2	3	98
A389: Ketamine	1	2	3	98

ASK THE HEALTH WORKER TO SHOW YOU WHERE DELIVERY EQUIPMENT IS STERILIZED.

EQUIPMENT USED FOR STERILIZATION	Availability				If Not seen, not available,	Functioning *		
	Observed	Reported not seen	Not available	Don't know		Yes	No	DK

					**DK**			
A390: Electric autoclave (Pressure/Wet Heat)	1	2	3	98	Go to →A391	1	2	98
A391: Non-electric autoclave (Pressure/Wet Heat)	1	2	3	98	Go to →A392	1	2	98
A392: Electric dry heat sterilizer	1	2	3	98	Go to →A393	1	2	98
A393: Electric boiler or steamer (no pressure)	1	2	3	98	Go to →A394	1	2	98
A394: Non-electric pot with cover (for steam/boil)	1	2	3	98	Go to →A395	1	2	98
A395: Heat source for non-electric equipment	1	2	3	98	Go to →A396	1	2	98
A396: Automatic Timer (May be on equipment)	1	2	3	98	Go to →A397	1	2	98
A397: TST Indicator strips or other item that indicates when sterilization is complete.	1	2	3	98	Go to →A398	1	2	98
A398: Chlorine-based or glutaraldehyde solution (for chemical method)	1	2	3	98				
A399: Written protocols or guidelines for sterilization or disinfection	1	2	3	98				
**END OF SECTION 3**								

Section 4: Antenatal Care Readiness

ASK TO SPEAK WITH THE HEAD OF ANTENATAL CARE UNIT (THIS MAY BE DIRECTOR/IN-CHARGE IF NO HEAD OF UNIT)

Question	Yes	No	DK	Go to
A400: Does this facility offer routine antenatal services?	1	0		A400 is No → END SECTION

ASK TO SEE THE PLACE WHERE ANTENATAL CLIENTS ARE SEEN BEFORE THEY HAVE THEIR MEDICAL CONSULTATION AND INDICATE WHICH OF THE FOLLOWING ACTIVITIES ARE ROUTINELY CARRIED OUT THERE. OBSERVE IF THE BELOW ACTIVITIES ARE BEING CONDUCTED ROUTINELY. IF NOT SEEN ASK:

Question	Observed	Reported not seen	No	Don't know	Go to
A401: Is [*read activity*] routinely conducted for all antenatal care clients?					
A401a: Weighing clients	1	2	3	98	
A401b: Taking blood pressure	1	2	3	98	
A401c: Urine test for protein	1	2	3	98	
A401d: Blood test for anemia	1	2	3	98	
A401e: Tetanus toxoid vaccination	1	2	3	98	
A401f: Conducting group health education sessions	1	2	3	98	
A402: Which of the following activities are performed as part of routine services, that is, each client has this test at least once:	1	2	3	98	
A402a: Blood test for anemia	1	2	3	98	
A402b: Blood test for syphilis	1	2	3	98	
A402c: Blood group	1	2	3	98	
A402d: Test for Rh factor	1	2	3	98	
A402e: Urine test for protein	1	2	3	98	

	Yes	No	Don't know	Go to
A403: Which of the following types of services are routinely offered to antenatal clients:				
A403a: Preparing a birth plan/complication readiness	1	0	98	No → A403b
A403a1: Is it indicated on ANC card whether the family has a birth preparedness plan?	1	0	98	
A403b: Counseling about postpartum family planning	1	0	98	No → A404
A403b1: Is it indicated on ANC card the PPFP method client has chosen?	1	0	98	

THERE ARE NO MORE QUESTIONS FOR THE HEAD OF ANTENATAL CARE UNIT/DIRECTOR. EXPLAIN THAT FOR THE NEXT SECTION, YOU WILL NEED TO WALK AROUND AND LOOK AT THE ANTENATAL CARE EXAMINATION AREA. THEY CAN NOW CHOOSE TO ACCOMPANY YOU FOR THE REST OF THE ASSESSMENT OR ATTEND TO OTHER BUSINESS. IF THEY DO NOT ACCOMPANY YOU, ASK IF A HEALTH WORKER INVOLVED IN ANTENATAL CARE CAN HELP YOU WITH THE NEXT PART OF THE ASSESSMENT.

ASK TO SEE THE ROOM WHERE EXAMINATIONS FOR ANTENATAL CLIENTS ARE CONDUCTED.

A404: Describe the setting of the ANC examination room:	Code			
Private room with visual and auditory privacy	1			
Non-private room with visual and auditory privacy	2			
Visual privacy only	3			
No privacy	4			
**Question**	**Yes**	**No**	**DK**	**Go to**
A405: Is there a toilet for client use near the ANC service delivery area?	1	0		No →A406
A405a: Is the toilet functioning?	1	0	98	

NOTE THE AVAILABILITY AND CONDITION OF THE FOLLOWING SUPPLIES, EQUIPMENT AND MEDICATIONS NEEDED FOR ANC SERVICES. ITEMS MAY BE IN THE ROOM WHERE ANC EXAMINATIONS TAKE PLACE OR AN ADJACENT ROOM. IF YOU DO NOT SEE AN ITEM, ASK THE HEALTH WORKER HELPING YOU TO SHOW YOU THE ITEM.

SUPPLIES AND EQUIPMENT IN ANC EXAMINATION ROOM	Observed	Reported not seen	Not available	Don't know	Go to
A406: Examination gloves	1	2	3	98	
A407: Sharps container	1	2	3	98	
A408: Alcohol hand rub	1	2	3	98	
A409: Waste receptacle with lid and liner	1	2	3	98	
A410: Soap for hand washing	1	2	3	98	
A411: Water for hand washing	1	2	3	98	Not available/DK→A413
A412: How is water being made available for use in the ANC service area today?	**Code**				
Piped	1				
Bucket with tap	2				
Bucket or basin	3				
Don't Know	98				

EQUIPMENT MAY BE IN EXAMINATION ROOM, AN ADJACENT ROOM, OR ROOM WHERE MEASURE IS TAKEN.

	AVAILABILITY					FUNCTIONING*		
EQUIPMENT AND TESTING SUPPLIES	Observed	Reported not seen	Not available	Don't know	If Not seen, not available, DK	Yes	No	DK
A413: Blood pressure apparatus	1	2	3	98	Go to →A414	1	2	98
A414: Stethoscope	1	2	3	98	Go to →A415	1	2	98
A415: Fetal stethoscope (Fetoscope)	1	2	3	98	Go to →A416	1	2	98
A416: Doppler	1	2	3	98	Go to →A417	1	2	98
A417: Adult weighing scale	1	2	3	98	Go to →A418	1	2	98

MEDICATIONS/ VACCINE	Observed	Reported not seen	Not available	Don't know	Go to
A418: Iron and/or folic acid	1	2	3	98	
A419: Tetanus toxoid vaccine	1	2	3	98	
A420: Mebendazole/Albendazole	1	2	3	98	

END OF SECTION 4

Section 5: Family Planning Services

ASK TO SPEAK WITH THE HEAD OF FAMILY PLANNING SERVICES (THIS MAY BE DIRECTOR/IN-CHARGE IF NO HEAD OF UNIT)

Question	Yes	No	DK	Go to
A500: Does this facility offer family planning services?	1	0		A500 is No→ END SECTION
A501: Who provides family planning counseling at this facility?				
A501a: Midwife	1	0	98	
A501b: Community midwife	1	0	98	
A501c: General physician/clinician	1	0	98	
A501d: Obstetrician	1	0	98	
A501e: Other specialist	1	0	98	
A501f: Community health worker	1	0	98	
A501g: Student (nurse, midwife, medical)	1	0	98	
A502: What family planning methods are offered by this facility?				
A502a: Male condoms	1	0	98	
A502b: Female condoms	1	0	98	
A502c: Oral contraceptive pills	1	0	98	
A502d: Intrauterine device (IUD)/Postpartum intrauterine device (PPIUD)	1	0	98	
A502e: Implants	1	0	98	
A502f: Female sterilization	1	0	98	
A502g: Male sterilization	1	0	98	
A502h: Injectable (e.g. Depo-Provera)	1	0	98	
A502i: Emergency Contraception	1	0	98	
A503: Where are family planning commodities stored?	**Code**			
Pharmacy	1			
Store room	2			
Cabinet in FP area	3			
Drawer in delivery suite	4			
A504: Are commodities locked?	1	0		No →506
A505: Who manages access to the commodities				
A505a: Midwife	1	0	98	
A505b: Nurse midwife	1	0	98	
A505c: General physician/clinician	1	0	98	
A505d: Obstetrician	1	0	98	
A505e: Other (specify) ______________________________________	1	0	98	
A506: Have you had a stock- out of any contraceptives in the past 3 months?	1	0		No →A508
A507: How long did the stock-out last?	**Code**			
<1 week	1			
2-3 weeks	2			
>3 weeks	3			
Don't know	4			

THERE ARE NO MORE QUESTIONS FOR THE HEAD OF FAMILY PLANNING SERVICES. EXPLAIN THAT FOR THE NEXT SECTION, YOU WILL NEED TO WALK AROUND AND LOOK AT THE FAMILY PLANNING SERVICE PROVISION AREA. THEY CAN NOW CHOOSE TO ACCOMPANY YOU FOR THE REST OF THE ASSESSMENT OR ATTEND TO OTHER BUSINESS. IF THEY DO NOT ACCOMPANY YOU, ASK IF A HEALTH WORKER INVOLVED IN FAMILY PLANNING SERVICES CAN HELP YOU WITH THE NEXT PART OF THE ASSESSMENT.

A508: Describe the setting of the family planning examination and procedure room:	**Code**
Private room with visual and auditory privacy	1
Non-private room with visual and auditory privacy	2
Visual privacy only	3
No privacy	4

NOTE THE AVAILABILITY AND CONDITION OF THE FOLLOWING SUPPLIES, EQUIPMENT AND MEDICATIONS NEEDED FOR FAMILY PLANNING SERVICES. ITEMS MAY BE IN THE ROOM WHERE EXAMINATIONS TAKE PLACE OR AN ADJACENT ROOM. IF YOU DO NOT SEE AN ITEM, ASK THE HEALTH WORKER HELPING YOU TO SHOW YOU THE ITEM.

SUPPLIES AND EQUIPMENT IN FAMILY PLANNING EXAMINATION ROOM	Observed	Reported not seen	Not available	Don't know	Go to
A509: Stethoscope	1	2	3	98	
A510: Sphygmomanometer	1	2	3	98	
A511: Bivalve speculum	1	2	3	98	
A512: Uterine sound	1	2	3	98	
A513: Long placental forceps	1	2	3	98	
A514: Ring forceps for cleaning	1	2	3	98	
A515: Tenaculum for grasping cervix	1	2	3	98	
A516: Scissors	1	2	3	98	
A517: Gally pot for antiseptic	1	2	3	98	
A518: Mosquito forceps straight	1	2	3	98	
A519: Mosquito forceps curved	1	2	3	98	
A520: Scalpel	1	2	3	98	
A521: Local anesthesia (lidocaine 1%)	1	2	3	98	
A522: 5 or 10 cc syringe and 1.5 needle	1	2	3	98	
A523: Sharps container	1	2	3	98	
A524: Surgical tape to close incision and bandage to wrap the incision	1	2	3	98	
A525: Waste receptacle with lid and plastic liner	1	2	3	98	
A526: Soap for hand washing	1	2	3	98	
A527: Running water for hand washing	1	2	3	98	Not available/DK→A529
A528: How is water being made available for use in the service area today?	**Code**				
Piped	1				
Bucket with tap	2				
Bucket or basin	3				
Don't Know	98				

MAY BE IN EXAMINATION ROOM OR AN ADJACENT ROOM.

	AVAILABILITY				
**COMMODITIES**	**Observed**	**Reported not seen**	**Not available**	**Don't know**	**Go to**
A529: Male condoms	1	2	3	98	
A530: Female condoms	1	2	3	98	
A531: Oral contraceptive pills	1	2	3	98	
A532: IUDs	1	2	3	98	
A533: Implants	1	2	3	98	
A534: Injectable hormones (e.g. Depo-provera)	1	2	3	98	
A535: Cycle beads	1	2	3	98	
A536: Emergency contraception pill packets	1	2	3	98	

END OF SECTION 5

Section 6: HMIS

ASK TO SPEAK WITH THE PERSON RESPONSIBLE FOR HEALTH INFORMATION SYSTEMS .THIS MAY BE A DATA MANAGER/HMIS OFFICER, FACILITY-IN-CHARGE OR ANOTHER SERVICE PROVIDER

Question	Yes	No	DK	Go to
A600: Does this facility regularly produce reports for the national Health Management Information System?	1	0	98	
A601: Does this facility regularly compile any reports containing health services information *(IF YES, ASK TO SEE COPY OF MOST RECENT REPORT)*	1	0	98	A600 is No and A601 is No→ END SECTION
A602: How frequently are these reports compiled:	**CODE**			
Monthly or more often	1			
Every 2-3 months	2			
Every 4-6 months	3			
Less often than every 6 months	4			
A603: Are facility reports analyzed for data quality?	1	0	98	No→604
A603a: Are reports analyzed for completeness?	1	0	98	
A603b: Are reports checked against data in registers?	1	0	98	
A603c: Is a report on data quality produced? *(IF YES, ASK TO SEE COPY OF MOST RECENT REPORT)*	1	0	98	
A604: Does the facility display any of the following in table, graph, chart or map form?				
A604a: Data related to maternal health	1	0	98	No→ A604b
A604a1: *IF YES,* is it up to date?	1	0	98	
A604b: Data related to newborn health	1	0	98	No→ A604c
A604A1: *IF YES,* is it up to date?	1	0	98	
A604c: Data related to family planning	1	0	98	No→ A605
A604c1: *IF YES,* is it up to date?	1	0	98	
A605: Do implementing NGOs and/or provincial health authorities provide feedback on reports?	1	0	98	No→ A608
A606: Does feedback provided generally include recommendations for action?	1	0	98	
A607: *IF YES,* what types of action oriented recommendations have been made based on HMIS data?				
A607a: Review strategy by examining service performance target and actual performance from month to month	1	0	98	
A607b: Review facility personnel responsibilities by comparing service targets and actual performance month to month	1	0	98	
A607c: Mobilization/shifting resources based on comparison by services	1	0	98	
A607d: Advocacy for more resources by showing gaps in ability to meet targets	1	0	98	
A608: Does the facility have a functional Maternal and Perinatal Death Surveillance Response mechanism?	1	0	98	
A609: Does the facility have a functional mechanism for recording and sharing outcomes of cases referred in and out?	1	0	98	

END OF SECTION 6

Section 7: FACILITY STATISTICS – SERVICES PROVIDED

ASK IF THE FOLLOWING REGISTERS ARE ROUTINELY USED AT THIS FACILITY

Question	Yes	No	DK	Go to
A700: Antenatal care register	1	0	98	
A701: Labor and delivery ward register	1	0	98	
A702: Maternity ward register	1	0	98	
A703: Newborn unit register	1	0	98	
A704: Operating theater register	1	0	98	
A705: Post abortion care register	1	0	98	
A706: Postnatal care register	1	0	98	
A707: Family planning register	1	0	98	
A708: Postpartum family planning register	1	0	98	
A709: Discharge register	1	0	98	
A710: Referral register (general)	1	0	98	
A711: Obstetric referral register	1	0	98	
A712: Mortality/death register	1	0	98	

ASK IF YOU CAN REVIEW THE ANC, LABOR AND DELIVERY, PNC, FAMILY PLANNING SERVICE STATISTICS FOR THE LAST CALENDAR YEAR. NOTE THE TOTAL NUMBER OF CASES/MONTH FOR EACH INDICATOR BELOW FOR EACH OF THE 12 MONTHS. WRITE ZERO WHEN NO SERVICES WERE PROVIDED AND A ZERO IS NOTED IN THE SUMMARY REPORT/REGISTER. WRITE 9999 IF THERE IS NO INFORMATION AVAILABLE FOR THAT MONTH OR THE DATA IS MISSING.

	A	B	C	D	E	F	G	H	I	J	K	L
Month Name	Hamal	Sawer	Jawza	Saratan	Asad	Sonbula	Mizaan	Aqrab	Qaws	Jadi	Dalwa	Hoot
**ANTENATAL CARE**												
A713: ANC caseload (all visits)												
A714: ANC caseload (new visits)												
**DELIVERIES**												
A715: Deliveries (total)												
If A715=0 for all months → A735												
A716: Spontaneous vaginal deliveries (normal, breech, face)												
A717: Deliveries with vacuum extraction												
A718: Forceps deliveries												
A719: Craniotomies/ embryotomi												

es												
A720: Cesarean surgeries												
A721: Laparotomie s (for ruptured uterus)												
A722: Obstetric referrals in												
A723: Obstetric referrals out												

	A	B	C	D	E	F	G	H	I	J	K	l
Month Name	Hamal	Sawer	Jawza	Saratan	Asad	Sonbula	Mizaan	Aqrab	Qaws	Jadi	Dalwa	Hoot
**DIRECT OBSTETRIC COMPLICATIONS**												
A724: Antepartum hemorrhage												
A725: Postpartum hemorrhage												
A726: Retained placenta												
A727: Prolonged/ obstructed labor												
A728: Ruptured uterus												
A729: Postpartum sepsis												
A730: Severe pre-eclampsia/eclampsia												
A731: Abortion complications (hemorrhage and/or sepsis)												
A732: Postabortion women discharged with a contraceptive method												
A733: Ectopic pregnancy												
A734: Other direct obstetric complications (e.g. PPROM, malpresentation, pre-term labor, post-term, cord prolapse)												
**POSTNATAL CARE**												
A735: PNC caseload (all visits)												
A736: PNC caseload (new visits)												
**FAMILY PLANNING AND POST-ABORTION CARE**												
A737: FP caseload (all visits)												
A738: FP caseload (new visits)												
A739: IUD insertion												
A740: IUD removal												
A741: Implant insertion												
A742: Implant removal												
**END OF SECTION 7**												

Section 8: SERVICE STATISTICS – CLIENT OUTCOMES

CONTINUE TO REVIEW THE LABOR AND DELIVERY REGISTER. NOTE THE TOTAL NUMBER OF CASES/MONTH FOR EACH INDICATOR BELOW FOR EACH OF THE LAST 12 MONTHS. NOTE THE NAME OF EACH MONTH. WRITE ZERO WHEN NO CASES WERE REPORTED AND A ZERO IS NOTED IN THE SUMMARY REPORT/REGISTER. WRITE 9999 IF THERE IS NO INFORMATION AVAILABLE FOR THAT MONTH OR THE DATA IS MISSING.

	A	B	C	D	E	F	G	H	I	J	K	L
Month Name	Hamal	Sawer	Jawza	Saratan	Asad	Sonbula	Mizaan	Aqrab	Qaws	Jadi	Dalwa	Hoot
**MATERNAL DEATHS – DIRECT OBSTETRIC CAUSES**												
A800: Antepartum hemorrhage												
A801: Postpartum hemorrhage												
A802: Retained placenta												
A803: Obstructed/ prolonged labor												
A804: Ruptured uterus												
A805: Postpartum sepsis												
A806: Severe pre-eclampsia/eclampsia												
A807: Abortion complications (hemorrhage and/or sepsis)												
A808: Ectopic pregnancy												
A809: Other maternal deaths due to direct causes (e.g. embolism, anesthesia)												
**MATERNAL DEATHS – INDIRECT CAUSES**												
A810: Malaria												
A811: Anemia												
A812: Hepatitis												
A813: Other indirect causes												
**MATERNAL DEATHS – UNKNOWN CAUSES (OR WHERE NO INFORMATION IS AVAILABLE ON CAUSE OF DEATH**												
A814: Unknown causes												
**NEWBORN OUTCOMES**												
A815: Low birth weight babies (<2.5 kg); live births only												
A816: Low birth weight babies (>2.5 kg); live births only												
A817: Fresh stillbirths ($\geq$ 2.5 kg)												
A818: Fresh stillbirths (<2.5 kg)												
A819: Macerated stillbirths												
A820: Stillbirths (unspecified weight and/or timing of fetal death)												
A821: Very early neonatal deaths (first 24 hours; $\geq$ 2.5 kg)												
A822: Very early neonatal deaths (first 24 hours; $\leq$ 2.5 kg)												
A823: Very early neonatal deaths (unspecified birth weight)												

END OF SECTION 8

Section 9: Maternal Death Review

A MATERNAL DEATH IS DEFINED AS THE DEATH OF A WOMAN DUE TO OBSTETRIC CAUSES DURING PREGNANCY OR WITHIN 42 DAYS OF THE COMPLETION OF A PREGNANCY. THIS FORM SHOULD BE COMPLETED IN EACH HEALTH FACILITY FOR THE LAST 3 MATERNAL DEATHS THAT OCCURRED IN THE PREVIOUS 12 MONTHS. YOU WILL PROBABLY IDENTIFY THE WOMEN THROUGH ONE OF THE REGISTERS. ASK FOR HER CHART/RECORD, PARTOGRAPH, AND ANY OTHER HELPFUL INFORMATION.

IF ONLY 2 WOMEN DIED DURING THE PREVIOUS 12 MONTHS, COMPLETE THE FORM FOR THOSE 2 CASES AND WRITE "NA" IN THE CASE 3 COLUMN. IF ONLY ONE WOMAN DIED, COMPLETE THE REVIEW FOR THAT CASE ONLY AND WRITE "NA" IN THE CASE 2 AND CASE 3 COLUMNS. IF THERE HAS BEEN NO MATERNAL DEATH, WRITE "NA" FOR ALL THREE CASES AND GO ON TO THE NEXT SECTION.

Question	Case 1	Case 2	Case 3
A900: Age of woman	__ __	__ __	__ __
Don't Know/No information	98	98	98
A901: Gestational age	**Code**	**Code**	**Code**
Weeks	__ __	__ __	__ __
Term	88	88	88
Don't Know/No information	98	98	98
A902: When did the woman die?	**Code**	**Code**	**Code**
During pregnancy/before delivery	1	1	1
During abortion/ectopic	2	2	2
During vaginal delivery	3	3	3
During cesarean delivery	4	4	4
After vaginal delivery	5	5	5
After cesarean delivery	5	5	5
Don't Know/No information	98	98	98
A903: Was the woman referred to this facility?	**Code**	**Code**	**Code**
No	0	0	0
Yes, from a health post/community health worker	1	1	1
Yes, from a health center (SHC, BHC, CHC)	2	2	2
Yes, from a public hospital	3	3	3
Yes, from a private hospital or clinic	4	4	4
Other *(Specify)* ________________________	5	5	5
Don't Know/No information	98	98	98
A904: Location of delivery	**Code**	**Code**	**Code**
Not applicable (no delivery)	0	0	0
At home	1	1	1
On the way to the health facility	3	3	3
In a health post	4	4	4
In a health center (SHC, BHC, CHC)	5	5	5
In a public hospital	6	6	6
In a private hospital	7	7	7
Other *(Specify)* ________________________	8	8	8
Don't Know/No information	98	98	98
A905: Type of delivery	**Code**	**Code**	**Code**
Abortion/ectopic	0	0	0
Vaginal	1	1	1
Assisted with vacuum extractor or forceps	3	3	3
Cesarean	4	4	4
Destructive delivery (e.g. craniotomy, embryotomy)	5	5	5
Laparotomy (uterine rupture)	6	6	6
Don't Know/No information	98	98	98

Question	Case 1		Case 2		Case 3	
A906: Primary cause of death *(Specify)*	______		______		______	
Don't Know/No information	98		98		98	
A907: Secondary cause of death *(Specify)*	______		______		______	
Don't Know/No information	98		98		98	
A908: Date and time of onset of labor						
MM/DD/YYYY	__ __ / __ __ / __ __ __ __		__ __ / __ __ / __ __ __ __		__ __ / __ __ / __ __ __ __	
HH:MM	__ __ :__ __		__ __ :__ __		__ __ :__ __	
AM/PM	__ __		__ __		__ __	
Don't Know/No information	98		98		98	
A909: Date and time of onset of complications						
MM/DD/YYYY	__ __ / __ __ / __ __ __ __		__ __ / __ __ / __ __ __ __		__ __ / __ __ / __ __ __ __	
HH:MM	__ __ :__ __		__ __ :__ __		__ __ :__ __	
AM/PM	__ __		__ __		__ __	
Don't Know/No information	98		98		98	
A910: Date and time of delivery						
MM/DD/YYYY	__ __ / __ __ / __ __ __ __		__ __ / __ __ / __ __ __ __		__ __ / __ __ / __ __ __ __	
HH:MM	__ __ :__ __		__ __ :__ __		__ __ :__ __	
AM/PM	__ __		__ __		__ __	
Don't Know/No information/ N/A (abortion/ectopic)	98		98		98	
A911: Date and time of death						
MM/DD/YYYY	__ __ / __ __ / __ __ __ __		__ __ / __ __ / __ __ __ __		__ __ / __ __ / __ __ __ __	
HH:MM	__ __ :__ __		__ __ :__ __		__ __ :__ __	
AM/PM	__ __		__ __		__ __	
Don't Know/No information	98		98		98	
A912: Did the woman receive any of the following life-saving services, treatments or interventions? *(If item not recorded, select "0")*	**Yes**	**No**	**Yes**	**No**	**Yes**	**No**
A912a: IV fluids	1	0	1	0	1	0
A912b: Plasma	1	0	1	0	1	0
A912c: Blood transfusion	1	0	1	0	1	0
A912d: Antibiotics	1	0	1	0	1	0
A912e: Oxytocics	1	0	1	0	1	0
A912f: Anticonvulsants	1	0	1	0	1	0
A912g: Manual removal of placenta	1	0	1	0	1	0
A912h: D&C/E&C	1	0	1	0	1	0
A912i: Manual vacuum aspiration	1	0	1	0	1	0
A912j: Forceps extraction	1	0	1	0	1	0
A912k: Vacuum extraction	1	0	1	0	1	0
A912l: Cesarean	1	0	1	0	1	0
A912m: Hysterectomy	1	0	1	0	1	0
A912n: Laparotomy	1	0	1	0	1	0
A912o: Oxygen	1	0	1	0	1	0
A912p: Antimalarials	1	0	1	0	1	0
A912q: Other treatment *(specify)*	1	0	1	0	1	0

A913: Outcome of the newborn(s)	**Code**		**Code**		**Code**	
N/A (abortion/ectopic) → A916	0		0		0	
Alive→ A916	1		1		1	
Dead	2		2		2	
One alive, one dead (twins)	3		3		3	
Don't Know/No information→ A916	98		98		98	
A914: Was it a stillbirth or early neonatal death?	**Code**		**Code**		**Code**	
Stillbirth(s)	0		0		0	
Early neonatal death(s)	1		1		1	
One stillbirth, one early neonatal death (twins)	2		2		2	
Don't Know/No information	3		3		3	
A915: Cause of newborn death	**Code**		**Code**		**Code**	
Prematurity-related	0		0		0	
Asphyxia	1		1		1	
Infection/pneumonia	2		2		2	
Congenital anomalies	3		3		3	
Other *(specify)* ________________________	4		4		4	
Don't Know/No information	98		98		98	
A916: Factors that contributed to the maternal death *(Respond to the best of your ability, given the information available from providers or records and based on your own judgement)*	**Yes**	**No**	**Yes**	**No**	**Yes**	**No**
A916a: Delayed arrival to health facility	1	0	1	0	1	0
A916b: Delayed transfer to appropriate level of care	1	0	1	0	1	0
A916c: Delay in health facility due to lack of supplies (drugs, blood, etc)	1	0	1	0	1	0
A916d: Delay in health facility due to absence of or slowness of human resources	1	0	1	0	1	0
A916e: Delay in correct diagnosis	1	0	1	0	1	0
A917: *PLEASE ADD ANY ADDITIONAL COMMENTS ON THE CASES REVIEWED BASED ON INFORMATION AVAILABLE FROM PROVIDERS OR RECORDS AND BASED ON YOUR OWN JUDGEMENT*						

END OF SECTION 9. END OF FACILITY INVENTORY AND RECORD REVIEW.

AFGHANISTAN MNH QUALITY OF CARE FACILITY ASSESSMENT

Tool B: Matneral and Newborn Health Worker Interview and Knowledge Test

Health facility visited (name):						
Health facility code (from HMIS and/or facility listing):						
Health worker code (assigned during listing):						

TYPE OF HEALTH FACILITY	
Specialized hospital .... [1]<br>Regional hospital .... [2]<br>Provincial hospital .... [3]<br>District hospital .... [4]<br>Comprehensive health center .... [5]<br>Basic health center .... [6]<br>Sub health center .... [7]	Private hospital ....[8]<br>Private clinic ....[9]<br>Other (specify) ....[10]<br>_______________________________________
Province Name:	
District Name:	
City / Village Name:	
Name of Observer:	
Name of Team Leader	
Date of Visit: (dd/mm/yy)	__ __/ __ __/ __ __ __ __
Time of Visit: (hh:mm/am-pm)	__ __ :__ __/ __ __
Signature of Team Leader:	

B001: Facility name	B002: Facility number
B003: Observer number	B004: Today's date (day/month/year)

EXPLAIN TO THE HEALTH WORKER THAT HIS/HER NAME WAS PROVIDED AS A KNOWLEDGEABLE MATERNAL AND/OR NEONATAL HEALTH PROVIDER AVAILABLE ON THAT DAY. VALIDATE WITH THE HEALTH WORKER THAT HE/SHE DOES PROVIDE SOME MATERNAL AND/OR NEONATAL HEALTH SERVICES IN THIS FACILITY.

Hello, I am _________________. I am representing the Ministry of Public Health and the USAID-funded HEMAYAT Project. We are conducting a study of health facilities in this country, with the goal of finding ways to improve maternal and newborn health services. We are recruiting health care providers in more than 200 facilities across the country to participate in this study. Specifically, we are recruiting those who are providers of maternal and newborn health services. May I continue?

READ ORAL CONSENT SCRIPT TO HEALTH WORKER.

I would like to interview you and ask you some questions about your professional background and how to care for women during pregnancy and birth and their newborns. The interview should last about 45 minutes.

There may be no direct benefit to you from being in this study. Information from this interview is confidential. Your name will not be recorded. The information acquired during this interview may be used by the Ministry of Public Health or other organizations to improve services, or for research on health services; however, your name will not be entered into the database. You do not have to agree to be in this study, and you may change your mind at any time.

Do you have any questions for me? You may call the HEMAYAT Project Technical Director, Dr. Partamin, at 0700020686. You may also contact the the Ministry of Public Health Ethics Committee which approved this study by calling Dr. Sayed Murtaza Hofiani at 070055560 with any problems or concerns about the study**.**

Do you have any questions for me? If at any point you feel uncomfortable you can ask me to leave.

B005: *ASK HEALTH WORKER:* Do I have your permission to conduct this interview?

☐ Yes, consent is given → go to B006
☐ No, consent is not given → interview of this healthcare worker must <u>END</u>.

B006: Health worker line number (from staff listing):	B007: Sex of health worker<br>Male 0<br>Female 1
B008: Health worker category	**CODE**
Midwife or community midwife	1
Nurse or community health nurse	2
General physician/clinician	3
Obstetrician	4
Pediatrician	5
Other specialist	6
Community health worker	7
Traditional birth attendant	8
Student (nurse, midwife, medical)	9

Section 1: Interview

READ THE FOLLOWING QUESTIONS TO THE HEALTH WORKER. IF HEALTH WORKER DOESN'T KNOW THE YEAR, PROBE USING PAST EVENTS AND RECORD YOUR BEST ESTIMATE.

EDUCATION AND EXPERIENCE

Question	Code	
B100: What year did you graduate (or complete) with your qualification provided in the previous question?	☐☐☐☐	(*Use YYYY format)*
B101: In what year did you start working in your current position in this facility?	☐☐☐☐	
B102: What is your age? *(OBSERVER: IF HEALTH WORKER DOESN'T WANT TO GIVE AGE, ASK THEM TO GIVE YOU A RANGE, I.E. BETWEEN AGE 40 AND 50, AND THEN ENTER THE MIDDLE VALUE - 45)*	☐☐	

TRAINING AND SERVICES PROVIDED

Question	Yes	No	Go to
B103: In your current position, do you personally provide any antenatal services?	1	0	No→B105
B104: How many years in total have you provided such services? Service may have been here or in another facility *(Observer: enter 00 if less than 1 year of service)*	☐☐		
B105: During the past 3 years have you received any pre- or in-service training on subjects related to antenatal care?	1	0	No→B107
B106: In the past 3 years, did you receive any training on the following topics *(read each answer aloud)*:	1	0	
B106a. ANC screening (e.g., blood pressure, urine protein)	1	0	
B106b. Counseling for ANC (e.g., nutrition, Family planning and breastfeeding)	1	0	
B106c. Management of pre-eclampsia/eclampsia ，	1	0	
B106d. Other topic related to ANC (specify)_________________	1	0	
B107: In your current position, do you personally provide any delivery services? By that, I mean conducting the actual delivery of newborns.	1	0	No→B111
B108: How many years in total have you provided such services? Service may have been here or in another facility. *(Observer: enter 00 if less than 1 year of service)*	☐☐		
B109: How often do you use a partograph to monitor and manage labor fully? *(read each answer except for "don't know" and "no response" aloud)*:	**CODE**		
Never	1		
Rarely	2		
Sometimes	3		
Most of the time	4		
Always	5		
Don't Know	98		
No response / Refused	99		
B110: How often do you use active management of the third stage of labor) during normal vaginal births *(read each answer except for "don't know" and "no response" aloud)*:	CODE		
Never	1		
Rarely	2		
Sometimes	3		
Most of the time	4		
Always	5		
Don't Know	98		
No response / Refused	99		
B111: During the past 3 years have you received any pre- or in-service training on subjects related to labour and delivery care?	1	0	No→B113

Question	Yes	No	Go to
B112: In the past 3 years, did you receive any training on the following topics *(read each answer aloud)*:			
B112a: Basic emergency obstetric and newborn care (BEmONC)	1	0	
B112b: Comprehensive emergency obstetric and newborn care (CEmONC)	1	0	
B112c: Respectful maternity care	1	0	
B112e: Use of chlorhexidine for cord care	1	0	
B112f: Use of misoprostol for prevention and/or management of postpartum hemmorhage			
B112g: Maternal death or near miss reviews/audits	1	0	
B112h: Postpartum family planning	1	0	
B112i: Quality improvement approaches	1	0	
B112j: HMIS data quality and use			
B112k: Gender and human rights	1	0	
B112l: Other (*specify):*_______________________________	1	0	
B113: In the past 3 years, were there any trainings that you wanted to attend but were not able to?	1	0	No→B114
B113a: Why were you unable to attend training?	**Code**		
Not selected	1		
No time	2		
Too far away	3		
Family or other obligations	4		
Couldn't afford it	5		
Other (specify) ____________________	6		
B114: In your current position, and as a part of your work for this facility, do you personally provide care for newborns?	1	0	Yes→B115

OBSERVER: IF PROVIDER DOES NOT PERSONALLY PROVIDE CARE FOR PREGNANT WOMEN, DURING LABOR & DELIVERY OR CARE FOR NEWBORNS, STOP THE SURVEY.

Question	Yes	No	Go to
B115: How many years in total have you provided such services? Service may have been here or in another facility *(Observer: enter 00 if less than 1 year of service)*	☐☐		
B116: During the past 3 years have you received any pre- or in-service training on subjects related to newborn care?	1	0	No→B118
B117: In the past 3 years, did you receive any training on the following topics *(read each topic aloud)*:			
B117a: Essential newborn care (e.g., cord care, warming, early and exclusive breastfeeding)	1	0	
B117b: Newborn resuscitation with bag and mask (e.g.Helping Baby Breathe HBB)	1	0	
B118: During the past 3 years have you received any pre- or in-service training on subjects related to postpartum family planning?	1	0	No→B120
B119: In the past 3 years, did you receive any training on the following topics *(read each topic aloud)*:			
B119a: Postpartum IUD insertion and/or removal	1	0	
B119b: Postpartum implant insertion and/or removal	1	0	

NOW I WOULD LIKE TO ASK YOU SOME QUESTIONS ABOUT SUPERVISION YOU HAVE PERSONALLY RECEIVED. THIS SUPERVISION MAY HAVE BEEN FROM A SUPERVISOR EITHER IN THIS FACILITY, OR FROM OUTSIDE THE FACILITY.

SUPERVISION			
**Question**	**Yes**	**No**	**Go to**
B120: Do you receive technical support or supervision in your work at this facility? If so, when was the most recent time?	**CODE**		
No, never supervised	0		→B123
Yes, in the last 3 months	1		
Yes, more than 3 months ago	2		
B121: How does your supervisor treat you? *(Read all options)*	**CODE**		

Very disrespectfully	1	
Disrespectfully	2	
Neutrally	3	
Respectfully	4	
Very respectfully	5	
Don't know/don't want to answer	98	
B122: The last time you were personally supervised, did your supervisor do any of the following *(read each aloud)*:		
B122a: Check your records or reports	1	0
B122b: Observe your work	1	0
B122c: Give you verbal feedback about how you were doing your job	1	0
B122d: Provide any written comment about how you were doing your job	1	0
B122e: Provide updates on administrative or technical issues related to your work	1	0
B122f: Discuss problems you have encountered	1	0
B122g: Participate in quality of care improvement activities	1	0

NOW I WOULD LIKE TO ASK YOU SOME QUESTIONS ABOUT WORKING CONDITIONS AND OPPORTUNITIES IN THIS FACILITY.

**WORKING CONDITIONS IN FACILITY**			
**Question**	**Yes**	**No**	**Go to**
B123: Do you currently have a written job description for your position?	1	0	No→B124
B123a: Do you have a copy of the job description?	1	0	
B124: Do you think that you have equal treatment and opportunities as your colleagues of the opposite sex			
B124a: in terms of training?	1	0	
B124b: in terms of professional advancement?	1	0	
B124c: in terms of time off?	1	0	
B124d: in terms of work schedule?	1	0	
B124e: in terms of work load?	1	0	

FOR QUESTION B125-126, <u>DO NOT</u> READ THE ANSWER CHOICES ALOUD. IF YOU ARE NOT SURE WHETHER AN ANSWER GIVEN BY HEALTH WORKER MATCHES THAT LISTED, PROBE FOR MORE DETAIL. IF THEY GIVE AN ANSWER THAT IS NOT LISTED, MOVE ON TO THEIR NEXT ANSWER. USE THE PROBE TO ENCOURAGE HEALTH WORKER TO GIVE 3 ANSWERS. IF THEY CANNOT GIVE AN ANSWER, OR GIVE ONLY ANSWERS THAT DO NOT APPEAR IN LIST, CIRCLE DON'T KNOW.

**Question**	**Yes**	**No**	**Go to**
B125: Among the various things related to your working situation that you would like to see improved, can you tell me the three that you think would most improve your ability to provide good quality of care services? (*PROBE: Anything else?)*			
B125a: More support from supervisor	1	0	
B125b:More knowledge/ updates / training	1	0	
B125c: More supplies/drugs	1	0	
B125d: Better quality equipment / supplies	1	0	
B125e: Less workload (more staff)	1	0	
B125f: Better working hours / flexible times	1	0	
B125g: More incentives (salary, promotion, holidays)	1	0	
B125h: Increased security	1	0	
B125i: Better facility infrastructure	1	0	
B125j: Better facility linkages for transport	1	0	
B125k: Better facility linkages for referral	1	0	
B125l: More autonomy / independence	1	0	
B125m: Psychosocial support for staff	1	0	
B125n: More / better supervision	1	0	
B125o: More job aids / guidelines / standards	1	0	
B125p: Mechanism for reporting complaints or harrassment	1	0	
B125q: Don't know / None of these	1	0	

B126: Are there aspects of your life outside work that interfere with your ability to do your job? (*PROBE:* Anything else?)		
B126a: Childcare responsibilities	1	0
B126b: Household responsibiltiies	1	0
B126c: Lack of transport	1	0
B126d: Safety in getting to/from work	1	0
B126e: Lack of support from spouse or other family members	1	0
B126f: Don't know / None of these	1	0
END OF SECTION 1		

Section 2: Maternal Health Knowledge

FOR THE FOLLOWING QUESTIONS, READ THE QUESTION ALOUD TO THE HEALTH WORKER. DO NOT READ THE ANSWER CHOICES ALOUD. IF YOU ARE NOT SURE WHETHER AN ANSWER GIVEN BY HEALTH WORKER MATCHES THAT LISTED, PROBE FOR MORE DETAIL. IF THEY GIVE AN ANSWER THAT IS NOT LISTED, MOVE ON TO THEIR NEXT ANSWER. USE THE PROBE TO ENCOURAGE HEALTH WORKER ***TO GIVE AS MANY ANSWERS AS THEY CAN THINK OF****. IF THEY CANNOT GIVE AN ANSWER, OR GIVE ONLY ANSWERS THAT DO NOT APPEAR IN LIST, CIRCLE DON'T KNOW.*

READ ALOUD: PLEASE ANSWER THE FOLLOWING QUESTIONS ON MATERNAL HEALTH TO THE BEST OF YOUR KNOWLEDGE. MOST OF THE QUESTIONS I ASK YOU WILL REQUIRE MULTIPLE RESPONSES FROM YOU. ASSUME ALL NEEDED SUPPLIES, MEDICATIONS, AND EQUIPMENT ARE AVAILABLE. WHEN THINKING ABOUT YOUR ANSWERS, YOU SHOULD INCLUDE ACTIONS OR INTERVENTIONS THAT COULD BE DONE AT YOUR FACILITY AND AT A REFERRAL FACILITY. I WILL PROBE SOMETIMES TO HELP YOU REMEMBER SOME MORE INFORMATION. PLEASE PROVIDE ALL RESPONSES THAT COME TO MIND.

Question	Mentioned	Not mentioned	Go to
B200: What are the primary aspects of focused antenatal care? *PROBE*: Anything else?			
B200a: Minimum of four consultaitons	1	0	
B200b: Ensure woman has birth plan	1	0	
B200c: Prevent illness and promote health (tetanus toxoid vaccine, iron tablets, etc)	1	0	
B200d: Detect existing illnesses and manage complications	1	0	
B200e: Teach danger signs of pregnancy, childbirth and the postpartum period	1	0	
B200f: Promote breastfeeding	1	0	
B200g: Other			
B200h: Don't know	1	0	
B201: Which women require a special care plan? *PROBE*: Anything else?			
B200a: Women who have had a cesarean	1	0	
B200b: Women with 5 or more past deliveires	1	0	
B200c: Interval <2 years or >5 years between pregnancies	1	0	
B200d: Previous stillbirth	1	0	
B200e: Previous neonatal death	1	0	
B200f: Previous assisted delivery (vacuum extraction, forceps)	1	0	
B200g: History of severe obstetric complications	1	0	
B200h: Previous obstetric fistula repair	1	0	
B200i: Other			
B200j: Don't know	1	0	
B202: For a woman in labor, what observations do you make as you monitor her progress? *PROBE*: Anything else?			
B202a: Fetal heartbeat	1	0	
B202b: Color of amniotic fluid	1	0	
B202c: Degree of molding	1	0	
B202d: Dilation of the cervix	1	0	
B202e: Descent of the head	1	0	
B202f: Uterine contractions	1	0	
B202g: Maternal blood pressure	1	0	
B202h: Maternal temperature	1	0	

B202i: Maternal pulse	1	0	
B202i: Urine output	1	0	
**Question**	**Mentioned**	**Not mentioned**	**Go to**
B203: What are the key steps for performing active management of the third stage of labor? *PROBE*: If health worker mentions uterotonic, ask when should uterotonic be given?			
B203a: Administration of a uterotonic immediately / within 1 minute of delivery	1	0	
B203b: Administration of a uterotonic after delivery of placenta and checking for second infant	1	0	
B203c: Controlled cord traction	1	0	
B203d: Check uterine tone and massage if soft	1	0	
B203e: Don't know	1	0	
B203f: Other (specify): ______________________________	1	0	
B204: What actions are appropriate for a woman who presents with, or develops heavy bleeding postpartum from atonic / uncontracted uterus? *PROBE*: Any other actions or interventions?؟			
B204a: Massage the fundus	1	0	
B204b: Empty the urinary bladder	1	0	
B204c: Give uterotonics IM or IV	1	0	
B204d: Perform bimanual compression of uterus	1	0	
B204e: Perform abdominal compression of aorta	1	0	
B204f: Start IV fluids	1	0	
B204g: Take blood for Hb, grouping and x-matching	1	0	
B204h: Refer to doctor or hospital	1	0	
B204i: Raise foot of bed	1	0	
B204j: Insert uterine balloon tamponade			
B204k: Don't know	1	0	
B204l: Other (specify): ______________________________	1	0	
B205: When should membranes be ruptured artificially by the provider?			
B205a: At start of second stage	1	0	
B205b: Immediately prior to delivery when they are bulging in vagina	1	0	
B205c: Routinely during active phase of labor	1	0	
B205d: As part of augmentation of first stage labor	1	0	
B205e: Upon admission for all women	1	0	
B205f: To check color of fluid / liqour when fetal distress is noted	1	0	
B205g: Not to be ruptured	1	0	
B205h: Don't know	1	0	
B205i: Other (specify): ______________________________	1	0	
B206: What actions do you think are most appropriate in managing a woman with severe pre-eclampsia at term? *PROBE*: Any other actions?			
B206a: Provide magnesium sulfhate	1	0	
B206b: Provide diazepam	1	0	
B206c: Provide anti-hypertensives	1	0	
B206d: Prepare to deliver within 24 hours	1	0	
B206e: Don't know	1	0	
B206f: Other (specify): ______________________________	1	0	

Question	Mentioned	Not mentioned	Go to
B207: Can you describe the signs of obstructed labor / dystocia? *PROBE:* Anything else?			
B207a: No advance of the presenting part despite strong uterine contractions	1	0	
B207b: Slow or no dilation of the cervix despite strong uterine contractions	1	0	
B207c: Fetal distress	1	0	
B207d: Maternal distress	1	0	
B207e: Bandi's ring	1	0	
B207f: Third degree molding / caput	1	0	
B207g: Fever	1	0	
B207h: Ruptured uterus	1	0	
B207i: Other (Please specify)	1	0	
B207j: Don't know	1	0	
B208: Can you explain the possible outcomes of obstructed labor / dystocia? PROBE: Anything else?			
B208a: Infection	1	0	
B208b: Ruptured uterus	1	0	
B208c: Fistula	1	0	
B208d: Stillbirth	1	0	
B208e: Asphyxia	1	0	
B208f: Newborn death	1	0	
B208g: Other (Please specify)	1	0	
B208h: Don't know	1	0	
B209: Please tell me which antibiotics you would give to a woman who is diagnosed with postpartum endometritis following a vaginal delivery? *PROBE*: Anything else?			
B209a: Ampicillin	1	0	
B209b: Gentamicin	1	0	
B209c: Metronidazole	1	0	
B209d: Other antibiotic	1	0	
B209e: Other (Please specify)	1	0	
B209f: Don't know	1	0	
B210: Please tell me about the protocols of medication administration. *PROBE*: Anything else?			
B210a: Rights of medication administration (Let Health worker to say)			
☐ ☐ B210a1 Give the right drug	1	0	
☐ ☐ B210a2 To the right patient	1	0	
☐ ☐ B210a3 In the right dose	1	0	
☐ ☐ B210a4 By the right route	1	0	
☐ ☐ B210a5 At the right time	1	0	
☐ ☐ B210a6 And with the right documentation	1	0	
B210b: Proper identification of patient at time of administration of medication	1	0	
B210c: Documentation of medication with proper timings	1	0	
B210d: Other (Please specify)	1	0	
B211: When you see a woman with complications from incomplete abortion, what do you do? *PROBE:* ANYTHING ELSE?			
B211a: Do a vaginal exam	1	0	
B211b: Assess vaginal bleeding	1	0	
B211c: Assess vital signs	1	0	
B211d: Begin IV fluids	1	0	
B211e: Begin antibiotics	1	0	
B211f: Do (manual/electric) vacuum aspiration	1	0	
B211g: Do dialation with curettage or evacuation	1	0	
B211h: Provide counseling	1	0	
B211i: Refer	1	0	
B211j: Other (Please specify)	1	0	
B212: What information do you give patients who were treated for an incomplete abortion? *PROBE:* Anything else?			
B212a: Information on how to prevent reproductive tract infection/HIV	1	0	

B212b: Informaion about when a woman can conceive again	1	0
B212c: Counseling on family planning and services	1	0
B212d: Refer for family planning methods	1	0
B212e: Information on social support	1	0
B212f: Information about the consequences of an unsafe abortion	1	0
B212g: Other (Please specify)	1	0
B213: When a woman presents as a survivor of rape, what do you do? *PROBE:* Anything else?		
B213a: Encourage her to report to policy	1	0
B213b: Facilitate filling out a police report	1	0
B213c: Counsel for pre and post HIV testing	1	0
B213d: Counsel about pregnancy prevention	1	0
B213e: Provide emergency contraception	1	0
B213f: Provide post-exposure prophylaxis for HIV	1	0
B213g: Request that she do urine, vaginal smear/swab, and/or blood exams	1	0
B213h: Refer	1	0
B213i: Other (Please specify)	1	0

READ ALOUD: NOW I WOULD LIKE TO PRESENT YOU WITH A SCENARIO YOU MIGHT ENCOUNTER IN YOUR PRACTICE. A WOMAN IS BROUGHT TO THE EMERGENCY DEPARTMENT OF THE DISTRICT HOSPITAL BY HER HUSBAND AFTER SHE COMPLAINED OF A SEVERE HEADACHE AND BLURRED VISION. SHE IS 20 YEARS OLD, THIS IS HER FIRST PREGNANCY, AND SHE IS 37 WEEKS GESTATION. SHE HAD 2 ANC VISITS AND NO PROBLEMS. FETAL MOVEMENT IS NORMAL. HER BP IS 160/120. SHE HAS CONTRACTIONS 2 IN 10 MINUTES, LASTING 20 SECONDS BY PALPATION. HER URINE HAS 3+ PROTEIN.

B214: Given the information presented above, what is your working diagnosis? (do not read answers aloud and circle only 1 answer)	**CODE**
Kidney infection	1
Severe pre-eclampsia	2
Malaria	3
Eclampsia	4
In labor	5
Don't know	98

FOR QUESTION B215, READ THE QUESTION ALOUD TO THE HEALTH WORKER AND THEN READ EACH PROCEDURE ALOUD.

**Question** **Mulitple choice**	**Yes**	**No**	**D/K**
B215: Of the list of procedures I am going to read you, please tell me which procedures are carried out routinely for all patients during labor and delivery at your facility:	1	0	98
B215a: Artificial rupture of membranes	1	0	98
B215b: Active management of third stage of labor	1	0	98
B215c: Episiotomy in primigravida	1	0	98
B215d: Perineal shaving	1	0	98
B215e: Maternal blood pressure monitoring	1	0	98
B215f: Administration of prophylactic antibiotics to women in labor	1	0	98
B215g: Enema	1	0	98
B215h: Suctioning nose and mouth of newborn at time of birth	1	0	98
B215i: Intermittent fetal heart rate monitoring (approx. every hour or more often)	1	0	98

END OF SECTION 2

FOR THE FOLLOWING QUESTIONS, READ THE QUESTION ALOUD TO THE HEALTH WORKER. DO NOT READ THE ANSWER CHOICES ALOUD. IF YOU ARE NOT SURE WHETHER AN ANSWER GIVEN BY HEALTH WORKER MATCHES THAT LISTED, PROBE FOR MORE DETAIL. IF THEY GIVE AN ANSWER THAT IS NOT LISTED, MOVE ON TO THEIR NEXT ANSWER. USE THE PROBE TO ENCOURAGE HEALTH WORKER TO GIVE AS MANY ANSWERS AS THEY CAN THINK OF. IF THEY CANNOT GIVE AN ANSWER, OR GIVE ONLY ANSWERS THAT DO NOT APPEAR IN LIST, CIRCLE DON'T KNOW.

READ ALOUD: PLEASE ANSWER THE FOLLOWING QUESTIONS ON NEWBORN HEALTH TO THE BEST OF YOUR KNOWLEDGE. MOST OF THE QUESTIONS I WILL BE ASKING YOU WILL REQUIRE MULTIPLE RESPONSES FROM YOU. ASSUME ALL NEEDED SUPPLIES, MEDICATIONS, AND EQUIPMENT ARE AVAILABLE. WHEN THINKING ABOUT YOUR ANSWERS, YOU SHOULD INCLUDE ACTIONS OR INTERVENTIONS THAT COULD BE DONE AT YOUR FACILITY AND AT A REFERRAL FACILITY. I WILL PROBE SOMETIMES TO HELP YOU REMEMBER SOME MORE INFORMATION. PLEASE PROVIDE ALL RESPONSES THAT COME TO MIND.

SECTION 3: NEWBORN HEALTH KNOWLEDGE QUESTIONS

Question	Mentioned	Not mentioned	Go to
B301: What basic equipment and supplies must be available to ensure any baby receives appropriate immediate care after birth?			
B301a: Dry warm towels or cloths	1	0	
B301b: Sterile blade or scissors	1	0	
B301c: Sterile or disposable cord ties / clamps	1	0	
B301d: Cap for baby	1	0	
B301e: Source of warmth: heating lamp or incubator	1	0	
B301f: Self-inflating ventilation bag	1	0	
B301g: Newborn face mask size 1	1	0	
B301h: Newborn face mask size 0	1	0	
B301i: Mucus extractor / suction / bulb syringe	1	0	
B301j: Flat surface	1	0	
B301k: Clock or watch with seconds	1	0	
B301l: Don't know	1	0	
B302: Please tell me, when a baby is delivered and there is no complication, what care is important to give them immediately after birth and in the first hour? PROBE: Anything else?			
B302a: Ensure baby was breathing / crying	1	0	
B302b: Provide thermal protection (skin to skin)	1	0	
B302c: Bathe newborn shortly after birth	1	0	
B302d: Suction newborn	1	0	
B302e: Ensure mother initiates breastfeeding within 1 hour	1	0	
B302f: Assess / examine newborn within 1 hour	1	0	
B302g: Weigh newborn	1	0	
B302h: Provide eye prophylaxis / antibiotic ointment	1	0	
B302i: Give prelacteal feed / water	1	0	
B302j: Cut cord with sterile blade / scissors	1	0	
B302k: Other (Please specify)	1	0	
B302l: Don't know	1	0	
B303: Can you please tell me the signs and symptoms of severe infection (sepsis) in a newborn? *PROBE:* Any other signs or symptoms?			
B303a: Not able to feed/ stopped feeding well (attachment and suckling or finger test)	1	0	
B303b: High pitch cry	1	0	
B303c: Crackle sound while breathing	1	0	
B303d: Cyanosis	1	0	
B303e: Restless / irritability	1	0	
B303f: Fast breathing (with a timer / watch and count for 1 full minute)	1	0	
B303g: Chest in-drawing (exposed the chest and abdomen to look for chest movement)	1	0	

B303h: Temperature (to look for hypo or hyperthermia)	1	0
B303i: Convulsions or fits	1	0
B303j: Pus / redness around umbilicus	1	0
B303k: Skin pustules	1	0
B303l: No movement or movement only with stimulation	1	0
B303m: Yellow soles	1	0
B303n: Other (Please specify)	1	0
B303o: Don't know	1	0
B304: When a newborn weighs less than 2000 grams, what special care do you provide?		
B304a: Ensure the baby is warm with continuous/intermittent skin-to-skin with mother	1	0
B304b: Ensure the baby is warm by placing it in an incubator	1	0
B304c: Ensure the baby is warm by placing it in a radiant warmer	1	0
B304d: Provide extra support to the mother to establish breastfeeding	1	0
B304e: Assess for jaundice	1	0
B304f: Assess for breathing difficulties (need for O_2 supplementation)	1	0
B304g: Monitor baby for the first 24 hours	1	0
B304h: Ensure infection prevention	1	0
B304i: Other (Please specify)	1	0
B305: If you were to perform neonatal resuscitation on a baby who is not breathing and for whom back rubbing does not help, what are the steps you'd take, giving them to me in order?		
B305a: Call f or help	1	0
B305b: Explain condition of baby to the mother	1	0
B305c: Place the newborn face up	1	0
B305d: Wrap or cover baby, except for face and upper portion of chest	1	0
B305e: Position baby's head so neck is slightly extended	1	0
B305f: Start ventilation using bag and mask	1	0
B305g: Other (Please specify)	1	0
B306: What do you check the baby for during a postnatal check?		
B306a: Baby breastfeeding well	1	0
B306b: Proper positioning for breastfeeding	1	0
B306c: Color tone of baby	1	0
B306d: Temperture of baby	1	0
B306e: Difficulty breathing	1	0
B306f: Eye swelling or discharge	1	0
B306g: Umbilical cord	1	0
B306h: Baby's weight	1	0
B306i: Alertness of baby	1	0
B306i: Other (Please specify)	1	0
B307: What do you check the mother for during a postnatal check?		
B307a: Vaginal bleeding	1	0
B307b: Signs of infection (fever)	1	0
B307c: Blood pressure	1	0
B307d: Abdominal tenderness	1	0
B307e: Size and firmness of uterus	1	0
B307f: Deep vein thrombosis	1	0
B307g: Breast engorgement	1	0
B307h: Signs of anemia	1	0
B307i: Assess lochia (vaginal discharge)	1	0
B307j: Signs of depression	1	0
B307k: Dribbling urine	1	0
B307l: Cough or breathing difficulties	1	0
B307m: Other (Please specify)	1	0
**END OF SECTION 3**		

READ THE FOLLOWING QUESTIONS TO THE HEALTH WORKER.

SECTION 4: RESPECTFUL CARE			
**Question**	**Yes**	**No**	
B400: Is this facility equipped to allow for birth companions/family members to be present?	1	0	
B401: Is this facility equipped to allow women to deliver in a non-lithotomy (non-horizontal) position?	1	0	
B402: Do you think disrespect between health workers or cadres of health workers is an issue in this facility? (*If participant describes in detail disrespect that occurs at the facility, please document the specific information in H403*)	**CODE**		
Disrepespect is not an issue. Health workers are treated very respectfully	1		→B406
Disrespect is sometimes an issue. Some health workers are treated disrespectfully.	2		→ Document in B403
Disrespect is an issue. Health workers are treated disrespectfully.	3		→ Document in B403
Don't know	98		
B403: If different cadres are mentioned, please note (for example, "Doctors do not respect midwives").<br>Provide comment to explain:			
B404: Please describe how one might be disrespectful to a male health co-worker			
B405: Please describe how one might be disrespectful to a female health co-worker			
B406: Treating clients with respect and dignity during labor, birth, and after birth is a health workers duty.<br>406a: To what extent does the environment here allow you to provide respectful maternity care? Provide comment to explain:<br>406b: Are there aspects of the environment that limit your ability to provide respectful maternity care? Provide comment to explain:			

Question	Yes	No
B407: Have you or your co-workers experienced any of the following?		
B407a: Being yelled at, threatened or harassed at work	1	0
B407b: Being pushed, shaken or having objects thrown at you/them	1	0
B407c: Being slapped	1	0
B407d: Having arm twisted or hair pulled	1	0
B407e: Being punched with a fist or hit with something that hurt	1	0
B407f: Being kicked dragged or beaten	1	0
B407g: Being choked	1	0
B407h: Being threatened or attacked with a knife, gun or weapon	1	0
B407i: Being physically forced to have sexual intercourse	1	0
B407j: Being physically forced to perform other sexual acts	1	0

Question	Yes	No
B408: Have you observed or heard about clients experiencing any of the following?		
B408a: Being yelled at by facility staff	1	0
B408b: Being threatened by facility staff	1	0
B408c: Being harassed by facility staff	1	0
B408d: Being punched with a fist or hit with something that hurt by facility staff	1	0
B408e: Being kicked, dragged or beaten by facility staff	1	0
B408f: Being ignored by facility staff when they needed care	1	0

NOW I WOULD LIKE TO ASK YOUR PERSPECTIVE ON HEALTH SERVICES FOR MEN AND WOMEN. I WILL READ YOU SOME STATEMENTS. FOR EACH ONE, PLEASE TELL ME WHETHER YOU STRONGLY AGREE, AGREE, ARE NEUTRAL, DISAGREE OR STRONGLY DISAGREE.

B409: A woman who comes to the health facility for services without a companion should be treated the same as any other patient.	**CODE**
Strongly agree	1
Agree	2
Neither agree nor disagee/ neutral	3
Disagree	4
Strongly Disagree	5
Don't know	98
B410: It is appropriate and important for a husband to participate in MNCH services and FP counseling	**CODE**
Strongly agree	1
Agree	2
Neither agree nor disagee/ neutral	3
Disagree	4
Strongly Disagree	5
Don't know	98
B411: A woman should not choose a family planning method until she consults with her husband	**CODE**
Strongly agree	1
Agree	2
Neither agree nor disagee/ neutral	3
Disagree	4
Strongly Disagree	5
Don't know	98
B412: A woman who has not had a boy child should not be encouraged to use family planning	**CODE**
Strongly agree	1
Agree	2
Neither agree nor disagee/ neutral	3
Disagree	4
Strongly Disagree	5
Don't know	98
B413: Male and female children should be provided the same attention and care	**CODE**
Strongly agree	1
Agree	2
Neither agree nor disagee/ neutral	3
Disagree	4
Strongly Disagree	5
Don't know	98

END OF SECTION 4. END OF INTERVIEW.

Tool C: ANC Observation Checklist

Health facility visited (name):						
Health facility code (from HMIS and/or facility listing):						
Health worker code (assigned during listing):						

TYPE OF HEALTH FACILITY	
Specialized hospital .... [1]<br>Regional hospital .... [2]<br>Provincial hospital .... [3]<br>District hospital .... [4]<br>Comprehensive health center .... [5]<br>Basic health center .... [6]<br>Sub health center .... [7]	Private hospital ....[8]<br>Private clinic ....[9]<br>Other (specify) ....[10]<br>____
Province Name:	
District Name:	
City / Village Name:	
Name of Observer:	
Name of Team Leader	
Date of Visit: (dd/mm/yy)	__ __/ __ __/ __ __ __ __
Time of Visit: (hh:mm/am-pm)	__ __ :__ __/ __ __
Signature of Team Leader:	

C001: Facility name	C002: Facility number
C003: Observer number	C004: Today's date<br>(day/month/year)

BEFORE OBSERVING THE CONSULTATION, OBTAIN PERMISSION FROM BOTH THE SERVICE PROVIDER AND THE CLIENT. MAKE SURE THAT THE PROVIDER KNOWS THAT YOU ARE NOT THERE TO EVALUATE HIM OR HER, AND THAT YOU ARE NOT AN "EXPERT" TO BE CONSULTED DURING THE SESSION.

Hello, I am _________________. I am a [midwife / physician] representing the Ministry of Public Health and the USAID-funded HEMAYAT Project. We are conducting a study of health facilities in this country, with the goal of finding ways to improve maternal and newborn health services. We are recruiting health care providers in more than 200 facilities across the country to participate in this study. Specifically, we are recruiting those who are providers of maternal and newborn health services. May I continue?

READ TO HEALTHCARE WORKER.

I would like to be present to observe the duration of your consultation with this client in order to understand how antenatal care services are provided in this facility.

There may be no direct benefit to you from being in this study but the findings will inform activities at this facility. Information from this observation is confidential. Neither your name nor that of the client will be recorded. The information acquired during this observation may be used by the Ministry of Public Health or other organizations to improve services, or for research on health services; however, neither your name nor the name of your clients will be entered into the database. You do not have to agree to be in this study, and you may change your mind at any time.

Do you have any questions for me? You may call the HEMAYAT Project Technical Director, Dr. Partamin, at 0700020686. You may also contact the Ministry of Public Health Ethics Committee which approved this study by calling Dr. Sayed Murtaza Hofiani at 070055560with any problems or concerns about the study.

Do you have any questions for me? If at any point you feel uncomfortable you can ask me to leave. However, we hope you will not mind our observing your consultation.

C005: *ASK HEALTH WORKER:* Do I have your permission to be present at this consultation?

☐ Yes, consent is given → go to C006

☐ No, consent is not given → assessment of this healthcare worker must END.

C006: Health worker line number (from staff listing):		C007: Sex of health worker<br>Male 0<br>Female 1
C008: Health worker category		**CODE**
	Midwife or community midwife	1
	Nurse or community health nurse	2
	General physician/clinician	3
	Obstetrician	4
	Pediatrician	5
	Other specialist	6
	Community health supervisor	7
	Community health workers	8
	Student (nurse, midwife, medical)	9

** NOTE: IF A CLIENT IS SEEN BY MORE THAN ONE SERVICE PROVIDER DURING THE VISIT, BE SURE TO OBTAIN PERMISSION FROM EACH PROVIDER.*

READ ORAL CONSENT SCRIPT TO CLIENT.

Good day. , I am ___________________. I am a [midwife / physician] representing the Ministry of Public Health and the USAID-funded HEMAYAT Project. We are conducting a study of health facilities in this country, with the goal of finding ways to improve maternal and newborn health services. I would like to be present to observe your antenatal care consultation in order to better understand how health services are provided in this facility. We expect your antenatal care consultation will last less than one hour.

Other patients in this facility are also being asked to participate. Please know that whether you decide to allow me to observe your visit is completely voluntary, and you may quit at any time. Whether you agree to participate or not will not affect the services you receive. While there are no direct benefits to you from being in this study, we expect your participation will help improve maternal and newborn health services in Afghanistan. We are not evaluating your antenatal care provider or this facility. Neither your name nor the date of services will be provided on any shared data, so your identity and any information about you will remain completely confidential. You do not have to agree to be in this study, and you may change your mind at any time.

Do you have any questions for me? You may call the HEMAYAT Project Technical Director**,** Dr. Partamin, at 0700020686. You may also contact Ministry of Public Health Ethics Committee which approved this study by calling Dr. Sayed Murtaza Hofiani at 070055560with any problems or concerns about the study.

If at any point you feel uncomfortable you can ask me to leave. However, we hope you won't mind our observing your consultation. Do you have any questions for me?

C009: *ASK CLIENT:* Do I have your permission to be present while you are receiving services today?

☐ Yes, consent is given → go to C010

☐ No, consent is not given → observation of this client must END; if available, approach another client for participation.

C010: Client Code	*START CLIENT CODE AT 1 FOR EACH NEW FACILITY VISITED*

**SECTION 1: INTRODUCTION AND HISTORY TAKING**	
C100: Record time observation started	__ __ __ __

RECORD WHETHER THE HEALTH WORKER CARRIED OUT THE FOLLOWING STEPS:

**Question**	**Yes**	**No**	**DK**	**Go to**
C101: Greets the client (and others present) in a friendly and respectful manner?	1	0	98	
C102: Asks client if she wants a family member or companion to participate in the consultation?	1	0	98	
C103: Asks the client's age?	1	0	98	
C104: Asks if the client is taking any medication?	1	0	98	
C105: If first visit, asks about the date of the last menstrual period? *(OBSERVER: SELECT 98 IF NOT FIRST VISIT)*	1	0	98	
C106: If first visit, calculates the gestational age and tells the client the estimated date of delivery	1	0	98	
C107: Asks about number of prior pregnancies	1	0	98	NO/DK→C109
C107a: Number of prior pregnancies *(RECORD WOMAN'S NUMBER OF PRIOR PREGNANCIES; ENTER 00 IF NO PREVIOUS PREGNANCIES)*	__ __			
C108: Discusses any of the following complications or experiences for <u>prior</u> pregnancies: *(SELECT ALL THAT APPLY)*				
C108a: Heavy bleeding during or after delivery	1	0	98	
C108b: Anemia	1	0	98	
C108c: High blood pressure	1	0	98	
C108d: High fever or infection	1	0	98	
C108e: Convulsions	1	0	98	
C108f: Multiple pregnancies (twins or above)	1	0	98	
C108g: Prolonged labor	1	0	98	
C108h: Cesarean delivery	1	0	98	
C108i: Assisted delivery (forceps, vacuum extractor)	1	0	98	
C108j: Prior neonatal death (death of baby less than 1 month old)	1	0	98	
C108k: Prior stillbirth (baby born dead that does not breathe or cry)	1	0	98	
C108l: Postpartum depression	1	0	98	
C109: Asks about or discusses any of the following for the <u>current</u> pregnancy: *(SELECT ALL THAT APPLY)*				
C109a: Vaginal bleeding	1	0	98	
C109b: Fever	1	0	98	
C109c: Severe headaches and/or blurred vision	1	0	98	
C109d: Convulsions or loss of consciousness	1	0	98	
C109e: Severe difficulty breathing	1	0	98	
C109f: Persistent cough for 2 weeks or longer	1	0	98	
C109g: Severe abdominal pain	1	0	98	
C109h Foul smelling discharge	1	0	98	
C109i: Medication the client is taking	1	0	98	
C109j: If there are any other problems the client is concerned about (Please specify: ______________________________)	1	0	98	
**END OF SECTION 1**				

RECORD WHETHER THE HEALTH WORKER CARRIED OUT THE FOLLOWING:

SECTION 2: TESTS AND TREATMENTS				
**Question**	**Yes**	**No**	**DK/NA**	**Go to**
C200: Washes his/her hands with soap or use sanitizer prior to examination?	1	0	98	
C201: Explains to the client about the examination	1	0	98	
C202: Performs any of the following procedures:				
C202a: Weighs the client	1	0	98	
C202b: Takes the client's blood pressure	1	0	98	
C202c: Checks for signs of anemia by examining conjunctiva or nail beds, and symptoms (difficulty breathing, excessive fatigue)	1	0	98	
C202d: Measures the client's abdomen for fundal height	1	0	98	
C202e: Palpates the abdomen to determine fetal presentation (OBSERVER: SELECT NOT APPLICABLE if < 28 weeks)				
C202f: Listens for fetal heartbeat (OBSERVER: SELECT NOT APPLICABLE if < 28 weeks)	1	0	98	
C203: Gives the client any of the following				
C203a: Prescription for iron or folic acid (IFA) or both	1	0	98	
C203b: Supply of iron or folic acid (IFA) or both	1	0	98	
C203c: Prescription for or injection of tetanus toxoid (TT)	1	0	98	NO/DK→C203d
C203c1: Explains the purpose of the TT injection	1	0	98	
C203d: Prescription for or supply of deworming medication	1	0	98	NO/DK→C204
C203d1: Explained the purpose of deworming	1	0	98	
C203d2: Explained how to take deworming medication	1	0	98	
C204: Did the provider maintain visual privacy for the client?	1	0	98	
**END OF SECTION 2**				

SECTION 3: COUNSELING AND OUTCOME				
**Question**	**Yes**	**No**	**DK**	**Go to**
C300: Did the health worker inform the client about progress of the pregnancy?	1	0	98	
C301: Did the health worker counsel the client in any of the following reasons to seek immediate medical care:				
C301a: Vaginal bleeding	1	0	98	
C301b: Convulsions	1	0	98	
C301c: Severe headaches with blurred vision	1	0	98	
C301d: Fever and is too weak to get out of bed	1	0	98	
C301e: Severe abdominal pain	1	0	98	
C301f: Fast or difficult breathing	1	0	98	
C301g: Persistent cough for two weeks or more	1	0	98	
C301h: Foul smelling discharge	1	0	98	
C302: Did the health worker discuss any of the following:				
C302a: Asked the client where she will deliver	1	0	98	
C302b: Advised the client to prepare for delivery (e.g. set aside money, arrange for emergency transportation)	1	0	98	
C302c: Advised the client to use a skilled health worker during delivery	1	0	98	
C302d: Asked the client who will be the decision-maker about her care	1	0	98	
C302e: Discussed with client what items to have on hand at home for birth (e.g. sterile blade, clean cloths, soap)	1	0	98	
C302f: Asked the client if she has identified a potential blood donor	1	0	98	
C302g: Asked the client if she has identified a birth companion of her choice	1	0	98	
C303: Did the health worker discuss nutrition and healthy eating during pregnancy?	1	0	98	
C304: Did the health worker discuss exclusive breastfeeding for 6 months?	1	0	98	

C305: Did the health worker discuss postpartum family planning?<br>C305a: Were PPFP IEC materials used?	1<br>1	0<br>0	98<br>98	No/DK→C306
C306: Did the health worker mention the health benefits of longer inter-pregnancy intervals/risks of short intervals?	1	0	98	
C307: Did the health worker discuss long-acting contraceptive methods that can be used for both spacing and limiting?<br>C307a: Discussed PPIUCD<br>C307b: Discussed PPimplant	1<br><br>1<br>1	0<br><br>0<br>0	98<br><br>98<br>98	No→C308
C308: Did the health worker discuss correct use of lactational amenorrhea as a temporary method ? *(Correct use of LAM requires exclusive or near exclusive breastfeeding while baby is less than six months, nursing at least every 6 hours, and menses has not yet returned)*	1	0	98	
C309: Did the health worker write on the client's health card? (*OBSERVER: CHOOSE DK IF NO CARD/BOOKLET*)	1	0	98	
C310: Did the health worker advise on when to return for next visit?	1	0	98	
C311: Did the health worker discuss the importance of at least four ANC visits?	1	0	98	
C312: Did the health worker speak using easy-to-understand language for the client?	1	0	98	
C313: Did the health worker ask whether the client had any questions?	1	0	98	
C314: Did the provider perform an ultrasound or refer client for an ultrasound?<br>C314a: For what indication?<br>C314a1: Concern about fetal development or wellbeing<br>C314a2: Client request<br>C314a3 Other (*Specify*) ___________________________________	1<br><br><br>1<br>1<br>1	0<br><br><br>0<br>0<br>0	98<br><br><br>98<br>98<br>98	No→C315
C315: RECORD THE TIME ANC CONSULTATION ENDED<br>(*OBSERVER: USE 24H CLOCK*)	[ ][ ][ ][ ]			
END OF SECTION 3. *REMEMBER TO THANK CLIENT FOR THEIR PARTICIPATION IN THE STUDY*				

AT THE END OF THE CONSULTATION, ASK THE HEALTH WORKER THE FOLLOWING QUESTIONS:

**SECTION 4: QUESTIONS FOR HEALTH WORKER**		
C400: How many weeks pregnant is the client?<br>(*OBSERVER: ENTER 98 FOR DON'T KNOW)*	[ ][ ]	
C401: Is this the client's 1st, 2nd, 3rd, 4th, or 5th or more ANC visit at this facility during this pregnancy?		
First visit	1	
Second visit	2	
Third visit	3	
Fourth visit	4	
Fifth or more visit	5	
Don't Know	98	
C402: Is this a referral or a routine ANC visit?		
Referral visit	1	
Routine visit	2	
C403: Is this the client's first pregnancy?<br>*(OBSERVER: ENSURE THAT THE ANSWER TO C107 AGREES WITH THE ANSWER TO THIS QUESTION:*		
No, not first pregnancy	0	
Yes, first pregnancy	1	
C404: Record the outcome of the consultation (what happened at the time the observation concluded)		
Client goes home	1	→C500
Client referred (same facility)	2	→C405
Client admitted (same facility)	3	→C405
Client referred to other facility	4	→C405
Don't Know	98	

C405: What is client's diagnosis?
**END OF SECTION 4**

**SECTION 5: ADDITIONAL OBSERVATIONS**	
C500: How many health workers did the client interact with during her visit in total?	☐☐

Question	YES	NO	DK	Go to
C501: Did you see a health worker shout, insult or threaten the woman at any time?	1	0	98	
C502: Did you see a health worker shout, insult or threaten the woman's family members or companions at any time?	1	0	98	

C503: *PLEASE COMMENT ON THE QUALITY OF CARE PROVIDED: WAS THE PREGNANT WOMAN TREATED RESPECTFULLY? INFORMED OF PROCEDURES? WAS THE SITUATION CHAOTIC OR CALM? WERE THERE ANY MAJOR DELAYS IN NEEDED TREATMENT? IF SO, FOR WHAT DRUGS / PROCEDURES AND WHY? WERE MULTIPLE HEALTH WORKERS INVOLVED? WHO WAS INVOLVED?*
**END OF SECTION 5. END OF ANC OBSERVATION.**

AFGHANISTAN MNH QUALITY OF CARE FACILITY ASSESSMENT

Tool D: Labor and Delivery Observation Checklist

Health facility visited (name):						
Health facility code (from HMIS and/or facility listing):						
Health worker code (assigned during listing):						

TYPE OF HEALTH FACILITY	
Specialized hospital .... [1]<br>Regional hospital ....[2]<br>Provincial hospital .... [3]<br>District hospital .... [4]<br>Comprehensive health center .... [5]<br>Basic health center .... [6]<br>Sub health center .... [7]	Private hospital ....[8]<br>Private clinic..[9]<br>Other (specify) ....[10]<br>____
Province Name:	
District Name:	
City / Village Name:	
Name of observer:	
Name of Team Leader	
Date of Visit: (dd/mm/yy)	__ __ / __ __ / __ __ __ __
Time of Visit: (hh:mm/am-pm)	__ __ :__ __ / __ __
Signature of Team Leader:	

Tool D: Labor and Delivery Observation Checklist

D001: Facility name	D002: Facility number
D003: Observer number	D004: Today's date (day/month/year)

BEFORE OBSERVING THE CONSULTATION, OBTAIN PERMISSION FROM BOTH THE SERVICE PROVIDER AND THE CLIENT. MAKE SURE THAT THE PROVIDER KNOWS THAT YOU ARE NOT THERE TO EVALUATE HIM OR HER, AND THAT YOU ARE NOT AN "EXPERT" TO BE CONSULTED DURING THE SESSION.

Hello, I am _________________. I am a [midwife / physician] representing the Ministry of Public Health and the USAID-funded HEMAYAT Project. We are conducting a study of health facilities in this country, with the goal of finding ways to improve maternal and newborn health services. We are recruiting health care providers in more than 200 facilities across the country to participate in this study. Specifically, we are recruiting those who are providers of maternal and newborn health services. May I continue?

READ TO HEALTHCARE WORKER.

I would like to be present to observe your care of this client in order to understand how delivery services are provided at this facility.

There will be no direct benefit to you from being in this study. Information from this observation is confidential. Neither your name nor that of the client will be recorded. The information acquired during this observation may be used by the Ministry of Public Health or other organizations to improve services, or for research on health services; however, neither your name nor the name of your clients will be entered into the database. You do not have to agree to be in this study, and you may change your mind at any time.

Do you have any questions for me? You may call the HEMAYAT Project Technical Director, Dr. Partamin, at 0700020686. You may also contact the Ministry of Public Health Ethics Committee which approved this study by calling Dr. Sayed Murtaza Hofiani at 070055560with any problems or concerns about the study.

Do you have any questions for me? If at any point you feel uncomfortable you can ask me to leave. However, we hope that you will not mind us observing your care of this client.

D005: *ASK HEALTH WORKER:* Do I have your permission to be present at during your care of this client?

☐ Yes, consent is given → go to D006

☐ No, consent is not given → assessment of this healthcare worker must END.

D006: Health worker line number (from staff listing):		D007: Sex of health worker<br>Male 0<br>Female 1
D008: Health worker category		**CODE**
	Midwife or community midwife	1
	Nurse or community health nurse	2
	General physician/clinician	3
	Obstetrician	4
	Pediatrician	5
	Other specialist	6
	Community health supervisor	7
	Community health workers	8
	Student (nurse, midwife, medical)	9

*READ ORAL CONSENT SCRIPT TO CLIENT. IF CLIENT IS INCAPACITATED, NEXT OF KIN OR FAMILY FRIEND ACCOMPANYING CLIENT MAY GIVE CONSENT **[SEE NEXT PAGE FOR CONSENT FORM FOR NEXT-OF-KIN]**. CONSENT FOR CLIENT CANNOT BE GIVEN BY HEALTH WORKER OR FACILITY-IN-CHARGE. CLIENT OR PROXY CONSENT MUST BE OBTAINED PRIOR TO START OF OBSERVATION.*

Good day. I am __________________. I am a [midwife / physician] representing the Ministry of Public Health and the USAID-funded HEMAYAT Project. We are conducting a study of health facilities to improve maternity health services in this country. I would like to be present to observe your care during labor and delivery and the care of your newborn in order to better understand how health services are provided in this facility.

Other clients in this facility are also being asked to participate. Please know that whether you decide to allow me to observe your visit is completely voluntary, and you may stop the observation at any time. Whether you agree to participate or not will not affect the services you receive. While there are no direct benefits to you planned, we expect your participation will help improve maternity health services. Neither your name nor the date of services will be provided on any shared data, so your identity and any information about you will remain completely confidential. You do not have to agree to be in this study, and you may change your mind at any time.

Do you have any questions for me? You may call the HEMAYAT Project Technical Director**,** Dr. Partamin, at 0700020686. You may also contact the Ministry of Public Health Ethics Committee which approved this study by calling Dr. Sayed Murtaza Hofiani at 070055560 with any problems or concerns about the study.

Do you have any questions for me?

D009: *ASK CLIENT:* Do I have your permission to be present while you are receiving services today?

☐ Yes, consent is given → go to D011

☐ No, consent is not given → observation of this client must <u>END</u>; if available, approach another client for participation.

READ ORAL CONSENT SCRIPT TO NEXT-OF-KIN [HUSBAND OR IMMEDIATE FAMILY MEMBER] FOR CONSENT TO OBSERVE CARE OF PATIENT UNABLE TO BE ASKED CONSENT DUE TO CONDITIONS SUCH AS BEING UNCONCIOUS.

Good day. I am __________________. I am a [midwife / physician] representing the Ministry of Public Health and the USAID funded HEMAYAT Project. We are conducting a study of health facilities to improve maternity health services in this country. I would like to be present to observe the care of [name of patient] during labor and delivery and the care of your newborn in order to better understand how health services are provided in this facility.

Other patients in this facility are also being asked to participate. Please know that whether you decide to allow me to observe [name of patient]'s care is completely voluntary, and you may stop the observation at any time. Whether you agree to participate or not will not affect the services [name of patient] receives. There are no foreseen risks for [name of patient] to participate in this study. While there are no direct benefits to participating in this study, we expect the findings will help inform activities at this facility. Neither your name, the name of you [wife/family member] nor the date of services will be provided on any shared data, so your identity, [name of patient]'s identity, and any information about her will remain completely confidential. You do not have to agree to be in this study, and you may change your mind at any time.

Do you have any questions for me? You may call the HEMAYAT Project Technical Director, Dr. Partamin, at 0700020686. You may also contact the Ministry of Public Health Ethics Committee which approved this study by calling Dr. Sayed Murtaza Hofiani at 070055560with any problems or concerns about the study.

Do you have any questions for me?

D010: *ASK NEXT-OF-KIN:* Do I have your permission to be present while you are receiving services today?
☐ Yes, consent is given → go to D011
☐ No, consent is not given → observation of this client must END; if available, approach another client for participation.

		**CODE**
D011: Who gave consent	Client	0
	Next of kin / family friend	1
D012: Client code	_____________________	*Start client code at 1 for the first client observed at a given facility.*

MOST CLIENTS WILL BE IN LABOR WHEN ADMITTED AND OBSERVATION STARTS, HOWEVER SOME CLIENTS MAY HAVE ALREADY DELIVERED AND COME TO THE FACILITY WHEN THEY ARE EXPERIENCED A COMPLICATION (E.G. PPH OR PE/E). ALSO, SOME CLIENTS MAY EXPERIENCE A COMPLICATION (PE/E) BEFORE GOING INTO LABOR AND BE TREATED IN LABOR WARD.

D013: Client is admitted for:		**CODE**	**GO TO**
	Labor and delivery	1	
	Complication	2	
D014: Client condition on arrival			
	Walked in unaided	1	
	Concious but assisted/carried	2	
	Unconcious	3	
	Other	95	Specify ___________________________________
D015: Where is client coming from?			
	Other health facility	0	
	ANC or labor ward at this facility	1	
	Home/someplace else	2	
D016: Does client have a referral slip with her?			
	No	0	
	Yes	1	
D017: Type of complication:			
	Severe Pre-eclampsia/ eclampsia	0	→ *GO TO SEVERE PE/E CHECKLIST (TOOL D1) TO BEGIN THE OBSERVATION*
	Postpartum hemorrhage	1	→ *GO TO PPH CHECKLIST (TOOL D2) TO BEGIN THE OBSERVATION*
	Other	95	Specify: _________________________

SECTION 1: INITIAL CLIENT ASSESSMENT				
**Question**	**Yes**	**No**	**DK/NA**	**Go to**
D100: Was this section observed?	1	0		No→D200
D101: Record time observation started (*Observer:* Please use 24 hr clock)				

RECORD WHETHER THE PROVIDER CARRIED OUT THE FOLLOWING STEPS AND/OR EXAMINATIONS: (SOME OF THE FOLLOWING STEPS MAY BE PERFORMED SIMULTANEOUSLY OR BY MORE THAN ONE PROVIDER)

INTRODUCTION AND HISTORY TAKING				
**Question**	**Yes**	**No**	**DK/NA**	**Go to**
D102: Greets the client (and others present) in a friendly and respectful manner	1	0	98	
D103: Encourages the women to have a support person present during labor and birth	1	0	98	
D104: Asks client (and support person if present) if she has any questions	1	0	98	
D105: Asks client about presence of danger signs	1	0	98	No/DK→D106
D105a: Vaginal bleeding	1	0	98	
D105b: Fever	1	0	98	
D105c: Severe headaches and/or blurred vision	1	0	98	
D105d: Convulsions or loss of consciousness	1	0	98	
D105e: Severe difficulty breathing	1	0	98	
D105f: Severe abdominal pain	1	0	98	
D105g: If there are other problems the client is concerned about	1	0	98	
D105h: Other (Please specify_________________________________)	1	0	95	
D106: Checks client card OR asks client her age, length of pregnancy (gestational age) , and parity	1	0	98	
**EXAMINATION**				
**Question**	**Yes**	**No**	**DK/NA**	**Go to**
D107: Washes hands with soap and water or uses alcohol hand rub before any initial examination	1	0	98	
D108: Uses curtains or other visual barriers for privacy during exam	1	0	98	
D109: Explains procedures to woman (and support person if present)	1	0	98	
D110: Takes client's temperature on admission	1	0	98	
D111: Takes client's pulse	1	0	98	
D112: Takes blood pressure	1	0	98	No/DK→D113
D112a: Take blood pressure in sitting or lateral position	1	0	98	
D112b: Take blood pressure with arm at heart level	1	0	98	
D113: Asks/notes amount of urine output	1	0	98	
D114: Orders urine test	1	0	98	
D115: Performs abdominal examination:	1	0	98	No/DK→D116
D115a: Checks fundal height with measuring tape	1	0	98	
D115b: Checks fetal presentation by palpation of abdomen	1	0	98	
D115c: Checks fetal heart rate with fetoscope/doppler/ultrasound	1	0	98	No/DK→D115d
D115c1: Records fetal heart rate	1	0	98	
D115d: Other (Please specify_________________________________)	1	0	95	
D116: Washes hands with soap and water	1	0	98	
D117: Performs vaginal examination	1	0	98	No/DK→D118
D117a: Wears sterile gloves for vaginal examination	1	0	98	
D117b: Respectfully informs woman before conducting vaginal examination	1	0	98	
D117c: Patient privacy maintained - uses curtains or other visual barriers for privacy during exams	1	0	98	
D117d: Informs woman of findings	1	0	98	

Question	Yes	No	DK	Go to
D118: Refers client for a cesarean section	1	0	98	No → Section 2
D118a: Reason for referral	**CODE**			
Prolonged or obstructed labor	1			
Severe Pre-eclampsia / eclampsia	2			
Antepartum hemorrhage	3			
Previous c-section scar	4			
Fetal distress	5			
Cord prolapse	6			
Multiple pregnancies	7			
Abnormal presentation	8			
Other	95 Specify: ___________________________			
END OF SECTION 1				

SECTION 2: INTERMITTENT OBSERVATION OF FIRST STAGE OF LABOR				
**Question**	**Yes**	**No**	**DK**	**Go to**
D200: Was this section observed?	1	0		No→D300
D201: Record time observation started (*Observer:* Please use 24 hr clock)	[ ][ ][ ][ ]			

RECORD WHETHER THE HEALTH WORKER CARRIED OUT THE FOLLOWING STEPS AND/OR EXAMINATIONS: (SOME OF THE FOLLOWING STEPS MAY BE PERFORMED SIMULTANEOUSLY OR BY MORE THAN ONE HEALTH WORKER)

PROGRESS OF LABOR				
**Question**	**Yes**	**No**	**DK**	**Go to**
D202: Explains to woman what will happen in labor (at least once)	1	0	98	
D203: Encourages woman to consume fluids/food during labor (at least once)	1	0	98	
D204: Encourages woman to empty bladder during labor (at least once)	1	0	98	
D205: Encourages/assists woman to ambulate and assume different positions during labor (at least once)	1	0	98	
D206: Uses partograph to monitor labor	1	0		No→D207
D207: Checks woman's blood pressure at least once	1	0	98	
D208: Checks fetal heart rate at least once	1	0	98	
D209: OBSERVER: Did the rupture of membranes occur naturally prior to delivery?	1	0	98	No →D210
D210: OBSERVER: Did a complication arise in the first stage of labor?	1	0	98	No →D211
D210a: If yes, what action was completed?	**Code**			
Cons.ult with specialist	1			
Refer to other facility for specialist	2			
Prepare for assisted delivery	3			
Prepare for Caeserean-section	4			
Other	95 Specify: ______________________			
D211: OBSERVER: Is a support person present in the room at some point during labor?	1	0	98	
D212: OBSERVER: Did the health worker give the woman (and support person if present) at least one update on status and progress of labor?	1	0	98	
D213: OBSERVER: Did the woman request some pain relief for her pain?	1	0	98	No →D214
D213a: If the woman requested pain relief, did the health worker help manage her pain	1	0	98	No →D214

EXAMINATION & PROCEDURES				
**Question**	**Yes**	**No**	**DK**	**Go to**
D214: Washes hands with soap and water or uses alcohol hand rub prior to any examination of woman?	1	0	98	
D215: Uses curtains or other visual barriers for privacy during exams, births, and procedures	1	0	98	
D216: Wears sterile surgical gloves	1	0	98	
D217: Drapes woman (one drape under buttocks, one over abdomen)	1	0	98	
D218: Explains procedures to woman (and support person if present) before proceeding	1	0	98	
D219: Total number of vaginal examinations performed *(OBSERVER: TO THE BEST OF YOUR ABILITY, UPDATE THE ANSWER TO THIS QUESTION DURING INTERMITTENT OBSERVATION OF FIRST STAGE OF LABOR)*	[ ][ ]			If 0 →D220
D219a: Time of first vaginal exam (observer: *use 24h clock*)	[ ][ ][ ][ ]			If only 1 exam →D220

D219b: Time of second vaginal exam: (observer: *use 24h clock*)	[ ][ ][ ][ ]			If only 2exams →D220
D219c: Time of third vaginal exam: (observer: *use 24h clock*)	[ ][ ][ ][ ]			If only 3exams →D220
D219d: Time of fourth vaginal exam: (observer: *use 24h clock*)	[ ][ ][ ][ ]			If only 4exams →D220
D219e: Time of fifth vaginal exam: (observer: *use 24h clock*)	[ ][ ][ ][ ]			If only 5 exams →D220
D219f: Time of sixth vaginal exam: (observer: *use 24h clock*)	[ ][ ][ ][ ]			
D219g: If other than midwife performed vaginal examinations, who and how many time performed the examinations:	1	0	98	If No/DK →D220
D219g1: Doctor:	[ ][ ]			
D219g2: Student:	[ ][ ]			
D219g3: Other Specify:	[ ][ ]			
D220: Augments labor with oxytocin	1	0	98	If No/DK →D221
D220a: Documents reason for augmentation	1	0	98	
D221: Inserts urinary catheter during labor	1	0	98	No/DK →D222
D221a: Catheter inserted with sterile gloves under sterile technique	1	0	98	
D222: Administers antibiotics	1	0	98	No/DK→D223
D222a: Record the time the antibiotic was administered	[ ][ ][ ][ ]			
D222b: What route was used for administration of antibiotics?	**CODE**			
Intramuscular	1			
Intravenous	2			
Oral	3			
Other	95	Specify: ____________________		
D222c: Why were antibiotics administered?				
Treatment for chorioamnionitis	1			
Management of pre-labor rupture of membranes	2			
Preparation for C-section	3			
Routine/prophylactic	4			
Don't know	98			
D222d: Which antibiotic was administered? (SELECT ALL THAT APPLY)	Yes	No		
D222d1: Penicillin	1	0		
D222d2: Ampicillin	1	0		
D222d3: Gentamicin	1	0		
D222d4: Metronidazole	1	0		
D222d5: Cephalosporin	1	0		
D222d6: Other (Please specify:___________________________)	1	0		
D222d7: Don't know	1	0		

PREPARATION FOR DELIVERY

CHECK TO SEE IF THE FOLLOWING EQUIPMENT AND SUPPLIES ARE LAID OUT IN PREPARATION FOR DELIVERY. IF SOME SUPPLIES ARE IN A BIRTH KIT, LOOK/ASK TO DETERMINE WHICH ITEMS ARE INCLUDED.

Question	Yes	No	DK	Go to
D223: Prepares uterotonic drug to use for AMTSL	1	0	98	No/DK→D224
D223a: Which drug:	**Code**			
Oxytocin	1			
Misoprostol	2			
Ergometrine	3			
D224: Timer (clock or watch with seconds hand)	1	0	98	
D225: Self-inflating ventilation bag (250 or 500 mL)	1	0	98	
D226: Newborn face mask size 0	1	0	98	

D227: Newborn face mask size 1	1	0	98	
D228: Suction bulb or mucus extractor	1	0	98	
D229: Individual Catheter (to be used with suction)	1	0	98	
D230: Suction machine – manual or electric	1	0	98	
D231: At least two cloths/blankets (one to dry; one to cover)	1	0	98	
D232: Cap/hat for the newborn	1	0	98	
D233: Disposable cord ties or clamps	1	0	98	
D234: Sterile scissors or blade	1	0	98	
D235: Was this woman referred for a C-section	1	0	98	No → D236
D235a: Reason for referral:	**CODE**			
Prolonged or obstructed labor	1			
Pre-eclampsia / eclampsia	2			
Antenatal hemorrhage	3			
Previous scar	4			
Fetal distress	5			
Cord prolapse	6			
Other	95	Specify: ________________________		
D236: Has the woman completed the first stage of labor?<br><br>*IF FIRST STAGE OF LABOR IS NOT COMPLETE, CHECK AND UPDATE ANSWERS IN THIS SECTION AGAIN 15-30 MINUTES LATER*	1	0		Yes→D300
END OF SECTION 2				

SECTION 3: CONTINUOUS OBSERVATION OF SECOND & THIRD STAGE OF LABOR				
**Question**	**Yes**	**No**	**DK**	**Go to**
D300: Was this section observed?	1	0		No→D400
D301: Record time observation started (*Observer:* Please use 24 hr clock)	☐☐☐☐			

RECORD WHETHER THE HEALTH WORKER CARRIED OUT THE FOLLOWING STEPS AND/OR EXAMINATIONS: (SOME OF THE FOLLOWING STEPS MAY BE PERFORMED SIMULTANEOUSLY OR BY MORE THAN ONE HEALTH WORKER).

PREPARATION FOR DELIVERY				
**Question**	**Yes**	**No**	**DK**	**Go to**
D302: Puts on clean protective clothing in preparation for birth (goggles, gown or apron) *(MARK YES IF NO CONTAMINATION)*	1	0	98	
D303: Washes hands with soap and water or uses alcohol hand rub before any examination of woman *(OBSERVER: MARK YES IF DONE PREVIOUSLY AND NO CONTAMINATION)*	1	0	98	
D304: Wears sterile surgical gloves *(OBSERVER: MARK YES IF DONE PREVIOUSLY AND NO CONTAMINATION)*	1	0	98	
D305: Performs episiotomy	1	0	98	No → D306
D305a: Reasons for doing episiotomy	**CODE**			
Fetal Distress	1			
Breech or face presentation	2			
Previous 3rd/4th degree tear	3			
Primigravida	4			
Other	5			
D305b: Informs woman of reason for episiotomy	1	0	98	
D306: Presentation of baby	**CODE**			
Cephalic	0			
Non-cephalic	1			

DELIVERY & UTEROTONIC				
**Question**	**Yes**	**No**	**DK**	**Go to**
D307: Delivers in the designated delivery room	1	0	98	
D308: As baby is delivered, health worker gently places on mothers abdomen on a clean cloth	1	0	98	
D309: Record time of the delivery of the baby:	☐☐☐☐			
D310: Second baby present? *(OBSERVER: MARK 1 IF MULTIPLE BABIES)*	1	0	98	No →D311
D310a: Record time of the delivery of second baby:	☐☐☐☐			
D311: Administers uterotonic?	1	0	98	No →D312
D311a: Record time uterotonic given:	☐☐☐☐			
D311b:Timing of administration of uterotonic	**CODE**			
Within 1 minute of delivery of baby	1			
Within 3 min of delivery of baby prior delivery of placenta	2			
More than 3 min after delivery of baby prior delivery of placneta	3			
After delivery of placenta	4			
D311c: Which uterotonic given	**CODE**			
Oxytocin	1			
Misoprostol	2			
Ergotmetrine	3			
D311d: Record dose of uterotonic given *(OBSERVER: IF NECESSARY, ASK)*	______________________________			

D311e: Route uterotonic given:<br>IM<br>IV<br>Oral<br>Other	CODE<br>1<br>2<br>3<br>95 Specify:___________________________
D312: Record the time the placenta was delivered:	☐☐☐☐<br>*(OBSERVER: Enter 98 if time not captured)*
D313: Health worker checks uterine tone immediately following the delivery of the placenta	1 0 98
D314: Assesses completeness of the placenta and membranes	1 0 98
D315: Assesses for perineal and vaginal lacerations	1 0 98
D316: OBSERVER: Did mother give birth in lithotomy position (on her back)	1 0 98
D317 OBSERVER: Is a support person (companion) for mother present at birth?	1 0 98
D318: OBSERVER: How many health workers and students are present at bedside at time of delivery?	☐☐
END OF SECTION 3	

SECTION 4: IMMEDIATE NEWBORN AND POSTPARTUM CARE				
**Question**	**Yes**	**No**	**DK**	**Go to**
D400: Was this section observed?	1	0		No→ D500

RECORD WHETHER THE HEALTH WORKER CARRIED OUT THE FOLLOWING STEPS AND/OR EXAMINATIONS: (SOME OF THE FOLLOWING STEPS MAY BE PERFORMED SIMULTANEOUSLY OR BY MORE THAN ONE HEALTH WORKER)

IMMEDIATE CARE				
**Question**	**Yes**	**No**	**DK**	**Go to**
D401: Immediately dries baby with towel	1	0	98	
D402: Is the baby breathing or crying?	1	0	98	No → SECTION 8 in form D3
D403: Places baby on mother's abdomen "skin to skin"	1	0	98	No / DK → D404
D403a: Covers baby including the head with dry towel	1	0	98	No→ D405
D404: Wraps baby in dry towel	1	0	98	
D405: Record the time the cord was tied or clamped:	☐☐☐☐ *(OBSERVER: Enter 98 if time not captured)*			
D405a: Was this at least 1 minute after birth (not immediately after birth)?	1	0	98	
D405b: Was cord cut with sterile blade or sterile scissors?	1	0	98	
D406: Applies 7.1% chlorhexidine gel to the cord stump	1	0	98	
D407: Mother informed of sex of baby	1	0	98	
D407a: Record sex of baby	**Code**			
Male	1			
Female	2			
**HEALTH CHECK**				
D408: Record time of health check	☐☐☐☐			
D409: Checks baby's temperature, by touch, 15 minutes after birth	1	0	98	
D410: Takes mother's vital signs 15 minutes after birth	1	0	98	
D411: Palpates uterus 15 minutes after delivery of placenta	1	0	98	

DURING FIRST HOUR AFTER BIRTH				
**Question**	**Yes**	**No**	**DK**	**Go to**
D412: OBSERVER: Are mother and newborn kept in same room after delivery (rooming-in)?	1	0	98	
D413: OBSERVER: Is baby kept skin to skin with mother?	1	0	98	
D414: Assists mother to start breastfeeding within first hour after birth	1	0	98	
D414a: Explains need for exclusive breastfeeding of baby on demand day and night fo r the first 6 months of life	1	0	98	
D415: Advises mother to stay in the facility for at least 6 hours	1	0	98	
D416: Conducts repair for episiotomy or laceration	1	0	98	No/DK→D417
D416a: Repair was done with sterile gloves and maintaining sterile technique	1	0	98	
D416b: Repair was done with local anesthesia (Lignocaine)	1	0	98	
D417: Counsels mother on postpartum family planning	1	0	98	No/DK→D419
D417a: Counsels mother on PPIUCD	1	0	98	
D418: OBSERVER: Does the mother request a PPIUCD?	1	0	98	No/DK→ D419
D418a: OBSERVER: Does the mother receive a PPIUCD?	1	0	98	

D419: Administers antibiotics to mother postpartum	1	0	98	No/DK→ D420
D419a: Why were antibiotics administered?	**Code**			
Treatment for chorioamnionitis	1			
Routine/prophylactic	2			
Third stage/postpartum procedure	3			
Don't know	98			
D419b: Which antibiotic was administered: (SELECT ALL THAT APPLY)				
D419b1: Penicillin	1	0		
D419b2: Ampicillin	1	0		
D419b3: Gentamicin	1	0		
D419b4: Metronidazole	1	0		
D419b5: Cephalosporin	1	0		
D419b6: Other (Specify):_______________	1	0		
D419b7: Don't know	1	0		
D419c: What was the dose of antibiotic administered?	_________________________			
D419d: What was the route of administrate of the antibiotic?	**CODE**			
IV	1			
IM	2			
Oral	3			
**AFTER BIRTH**				
RECORD WHETHER THE HEALTH WORKER CARRIED OUT THE FOLLOWING STEPS AND/OR EXAMINATIONS AT SOME POINT AFTER BIRTH: (SOME OF THE FOLLOWING STEPS MAY BE PERFORMED SIMULTANEOUSLY OR BY MORE THAN ONE HEALTH WORKER)				
**Question**	**Yes**	**No**	**DK**	**Go to**
D420: Provides tetracycline eye ointment 1% prophylaxis	1	0	98	
D421: Administers Vitamin K to newborn	1	0	98	
D422: Weighs baby and documents the weight	1	0	98	
D422a: OBSERVER: Record weight of the baby	[ ][ ][ ][ ]			
D422b: OBSERVER: Is baby weight < 2500gm?	1	0	98	NO / DK→ D423
D422c: Explains to the mother (and family members if present) that the baby has a low birth-weight and requires extra care	1	0	98	
D422d: Was baby transferred/referred to newborn unit?	1	0	98	YES→ D423
D422e: Explains need for continuous skin to skin care, and assists mother to place baby correctly	1	0	98	
D422f: Observes mother breastfeeding	1	0	98	
D422f1:Counsels and assists mother to breastfeed if needed				
D422f1:Demonstrates how to feed expressed breastmilk with a cup and spoon if baby does not suckle	1	0	98	
D422g: Explains danger signs that indicate a need for immediate care	1	0	98	
**CLEAN-UP AFTER BIRTH**				
RECORD WHETHER THE HEALTH WORKER CARRIED OUT THE FOLLOWING STEPS AND/OR EXAMINATIONS: (SOME OF THE FOLLOWING STEPS MAY BE PERFORMED SIMULTANEOUSLY OR BY MORE THAN ONE HEALTH WORKER)				
**Question**	**Yes**	**No**	**DK**	**Go to**
D423: Disposes of all sharps in a puncture-proof container immediately after use	1	0	98	
D424: Decontaminates all reusable instruments in 0.5% chlorine solution	1	0	98	
D425: Sterilizes or uses high-level disinfection for all reusable instruments	1	0	98	
D426: Disposes of all contaminated waste in leak-proof containers	1	0	98	
D427: Removes apron and wipe with chlorine solution	1	0	98	
D428: Washes his/her hands with soap and water or uses alcohol hand rub	1	0	98	
D429: **Record time L&D observation ended** (*OBSERVER: USE 24H CLOCK*)	[ ][ ][ ][ ]			

END OF SECTION 4

SECTION 5: OUTCOME & REVIEW OF DOCUMENTATION	
**Question**	**CODE**
D500: Record outcome for the mother	
Discharged to home immediately after delivery	1
Goes to postpartum ward	2
Referred to specialist, same facility	3
Goes to surgery, same facility	4
Referred, other facility	5
Death of mother	6
Other (i.e. stays in same room as delivery)	98 Specify: ___________________________
D501: Record outcome for the newborn or fetus	
Discharded immediately to home with mother	1
Goes to normal nursery	2
Referred to specialist, same facility	3
Referred, other facility	4
Goes to postpartum ward with mother	5
Stays in labor room with mother	6
Newborn death	7
Fresh stillbirth	8
Macerated stillbirth	9
Don't know	98

POTENTIALLY HARMFUL PRACTICES				
**Question**	**YES**	**NO**	**DK**	**Go to**
D502: Did you see any of the following harmful or inappropriate practices by health workers that are never indicated *(SELECT ALL THAT APPLY)*				
D502a: Use of enema	1	0		
D502b: Apply fundal pressure to hasten delivery of baby or placenta	1	0		
D502c: Lavage of uterus after delivery	1	0		
D502d: Stretching of the perineum	1	0		
D502e: Bathing of newborn within first hour after birth	1	0		
D503: Did you see any of the following practices done without an appropriate indication (SELECT ALL THAT APPLY)				
D503a: Restrict food and fluids in labor	1	0		
D503b: Manual exploration of the uterus after delivery	1	0		
D503c: Use of episiotomy	1	0		
D503d: Routine aspiration of newborn mouth and nose at birth	1	0		
D503e: Routine intravenous line started without indication	1	0		

DISRESPECTFUL OR ABUSIVE PRACTICES				
**Question**	**YES**	**NO**	**DK**	**Go to**
D504: Did you see a health worker do any of the following? *(SELECT ALL THAT APPLY)*				
D504a: Slap, hit or pinch the woman during labor or after	1	0		
D504b: Slap newborn	1	0		
D504c: Hold newborn upside down	1	0		
D504d Shout, insult or threaten the woman at any time	1	0		

REVIEW PARTOGRAPH AND / OR CLIENT RECORD FOR COMPLETENESS				
**Question**	**YES**	**NO**	**DK**	**Go to**
D505: Was there a newborn resuscitation? *(observer: check answer to D402)*	1	0		No→ D507
EXAMINE CHART TO DETERMINE WHETHER THE HEALTH WORKER RECORDED THE FOLLOWING INFORMATION:				
D505a: Condition of the newborn at birth	1	0		
D505b: Procedures necessary to initiate breathing	1	0		
D505c: Time from birth to initiation of spontaneous breathing recorded or time of death if unsuccessful recorded	1	0		
D505d: Any clinical observations during resuscitation, including baby vital signs	1	0		
D505e: Final outcome of resuscitation measures	1	0		
D506: Was the partograph used to monitor labor? (ASK TO SEE COMPLETED PARTOGRAPH)	1	0		No→ D507
D506a. Partograph seen	1	0	98	No → D507
D506b. Partograph initiated at 4cm or >4cm	1	0	98	

*FOR THE FOLLOWING QUESTIONS: EXAMINE PARTOGRAPH AND/OR CHART TO DETERMINE THE FOLLOWING INFORMATION. IF THE INFORMATION IS NOT IN THE CHART OR PARTOGRAPH, BUT YOU KNOW THE INFORMATION OR PREVIOUSLY RECORDED THE INFORMATION IN ANOTHER SECTION, FILL IN THE ANSWER. IF THE INFORMATION IN THE CHART OR PARTOGRAPH DIFFER FROM YOUR INFORMATION, USE INFORMATION FROM YOUR OBSERVATION .**(OBSERVER: ENTER 98 IF UNKNOWN)***

D507: Age of woman	[ ][ ]
D508: Gravidity of the woman	[ ][ ]
D509: Parity of the woman <u>prior to this delivery</u>	[ ][ ]
D510: Gestational age at birth in weeks	[ ][ ]
D511: Time of admission to labor ward	[ ][ ][ ][ ]
D512: Centimeters dilated upon admission to labor ward	[ ][ ]
D513: Time membranes ruptured	[ ][ ][ ][ ]
D514: Method of membrane rupture	**Code**
Spontaneous	1
Artificial	2
Don't know	98
D515: Type of delivery	**Code**
Spontaneous vaginal	1
Assisted (instrumented)	2
Caesarean	3
Don't know	98
D516: Time of birth	[ ][ ][ ][ ]
D517: Birth weight in grams	[ ][ ][ ][ ]

Question	YES	NO	DK	Go to
D518: Was the mother diagnosed with severe pre eclampsia or eclampsia?	1	0		No→D519
D518a: Was baby delivered within 24 hours of severe pre eclampsia diagnosis or within 12 hrs of eclampsia diagnosis?؟	1	0		
D519: Did the mother have blood loss more than 500mL?	1	0		No→D520
D519a: Was she diagnosed with postpartum hemorrhage (recorded notes and register)?	1	0		
D520: Did the mother develop a fever of 38° C or higher during labor?	1	0		No→D521
D520a: Was she diagnosed with chorioamnionitis during labor?	1	0		
D521: Were antibiotics administered to mother at any time?	1	0		No → D522
D521a: When were antibiotics administered? (SELECT ALL THAT APPLY)				
D521a1: 1st stage	1	0		
D521a2: 2nd stage	1	0		
D521a3: 3rd stage	1	0		
D521a4: Postpartum	1	0		
D521b: Why were antibiotics administered? (SELECT ALL THAT APPLY)				
D521b1: Treatment for chorioamnionitis	1	0		
D521b2: After prelabor rupture of membranes	1	0		
D521b3: Preparation for C-section	1	0		
D521b4: Routine/prophylactic	1	0		
D521b5: Third stage/postpartum procedure	1	0		
D521b6: Don't know	1	0		
D521c: Which antibiotic was administered? (SELECT ALL THAT APPLY)				
D521D1: Penicillin	1	0		
D521D2: Ampicillin	1	0		
D521D3: Gentamicin	1	0		
D521D4: Metronidazole	1	0		
D521D5: Cephalosporin	1	0		
D521D6: Other	1	0		
D521D7: Don't know	1	0		
D522: Did you observe a case of either maternal or neonatal sepsis?	1	0		No/DK→D523

D522a: *PLEASE COMMENT ON THE DETAILS OF THE CASE(S):*
What was the clinical management that was taken? What were your general observations of the situation?

D523: *PLEASE COMMENT ON THE QUALITY OF CARE PROVIDED THROUGHOUT LABOR AND DELIVERY:*
Was mother treated respectfully? Informed of procedures to herself and her baby? Was the situation chaotic or calm? Were there any major delays in needed treatment? If so, for what drugs/procedures and why? Were multiple health workers involved? Who? If maternal or newborn/fetal death occurred, describe the circumstances. Was the mother counseled about the death of newborn/fetus?

END OF SECTION 5 – MAKE SURE THAT D100 - D500 ARE COMPLETELY ANSWERED BEFORE MOVING ON TO NEXT CLIENT

AFGHANISTAN MNH QUALITY OF CARE FACILITY ASSESSMENT

Tool D1: Labor and Delivery Observation Complication Checklist: Severe PE/E

Health facility visited (name):						
Health facility code (from HMIS and/or facility listing):						
Health worker code (assigned during listing):						

TYPE OF HEALTH FACILITY	
Specialized hospital .... [1]<br>Regional hospital ....[2]<br>Provincial hospital .... [3]<br>District hospital .... [4]<br>Comprehensive health center................. [5]<br>Basic health center.................................. [6]<br>Sub health center .... [7]	Private hospital ....[8]<br>Private clinic...[9]<br>Other (specify) ....[10]<br>____
Province Name:	
District Name:	
City / Village Name:	
Name of observer:	
Name of Team Leader	
Date of Visit: (dd/mm/yy)	__ __ / __ __ / __ __ __ __
Time of Visit: (hh:mm/am-pm)	__ __ :__ __ / __ __
Signature of Team Leader:	

D001: Facility name	D002: Facility number
D003: Observer number	D004: Today's date (day/month/year)

**D600: Time observation began** (*OBSERVER: USE 24H CLOCK*)	☐☐☐☐

Question	YES	NO	DK	Go to
D601: Is the woman: (*SELECT ALL THAT APPLY*)				
D601a: Unconscious	1	0		
D601b: Convulsing	1	0		
D602: Cadre of health worker(s) giving care: (*SELECT ALL THAT APPLY*)				
D602a: Midwife or community midwife	1	0		
D602b: Nurse	1	0		
D602c: General physician/clinician	1	0		
D602d: Obstetrician	1	0		
D602e: Pediatrician	1	0		
D602f: Other specialist	1	0		
D602g: Community health supervisor	1	0		
D602h: Community health worker	1	0		
D602i: Student (Nurse, Midwife, Medical)	1	0		
D603: Is there a family member with the woman?	1	0	98	
D604: By reviewing the records, record the time diagnosis of severe preeclampsia/eclampsia was made: (*OBSERVER: USE 24H CLOCK, USE 99:99 IF NOT KNOWN*)	☐☐☐☐			
D605: By reviewing the records, record the blood pressure of the woman on admission (*USE 99:99 IF NOT KNOWN or NOT RECORDED)*	☐☐☐<br>-----------<br>☐☐☐			
D606: By reviewing the records, did the patient have proteinuria? Or recorded?	1	0		No→D607
D606a: Record the amount:	CODE			
Trace	1			
1+	2			
2+	3			
3+	4			
D607: Is the woman pregnant and not in labor?	1	0		Yes →D617
D608: Is the woman in labor?	1	0		Yes →D610
D609: Is the woman postpartum?	1	0		Yes →D613
**PREGNANT/IN LABOR**				
D610: Was labor induced?	1	0	98	
D611: Was labor augmented?	1	0	98	No → D613
D611a: Why was the labor augmented? *Please describe below.*				

D612: Was the woman referred for cesarean surgery?	1	0	98
**POSTPARTUM**			
D613: Date of the birth (*dd/mm/yyyy*)<br>D614: Time of the birth (*observer: use 24h clock, use 99:99 for DK*)	____/____/_______<br>[ ][ ][ ][ ]		
D615: Where was the baby delivered: *Please describe below.*			
D616: Type of delivery	**Code**		
Spontaneous vaginal	1		
Assisted (instrumental)	2		
Caesarean	3		
Don't know	98		

**MANAGEMENT/TREATMENT**			
D617: Was MgSO4 administered?	1	0	No→D618
D617a: Date of start of administration of MgSO4	____/____/_______ (*dd/mm*)		
D617b: Time of administration of MgSO4	[ ][ ][ ][ ]		
D617c: Amount of loading dose of MgSO4 (g)	____________		
D617d: Route of loading dose	1	0	
D616d1: IV	1	0	
D616d2: IM	1	0	
D616d3: Other	(specify):_______________		
D617e: Was a maintenance dose given as well?	1	0	No→D618
D617f: Route of maintenance dose			
D617f1: IV	1	0	
D617f2: IM	1	0	
D617f3: Other	(specify):_______________		
D617g: Amount of maintenance dose (gr):	________________		
D617h: Frequency of maintenance dose:	________________		
D618: Was diazepam administered?	1	0	No→D619
D618a: Time of administration of diazepam	[ ][ ][ ][ ]		
D618b: Dose of diazepam	____________		
D618c: Route of dose			
D618c1: IV	1	0	
D618c2: IM	1	0	
D618c3: Other	(specify):_______________		
D618d: Was a maintenance dose given as well?	1	0	No→D619
D618e: Route of maintenance dose			
D617e1: IV	1	0	
D617e2: IM	1	0	
D617e3: Other	(specify):_______________		
D618f: Amount of maintenance dose (mg):	________________		

D619: Was an antihypertensive administered?	1	0	No→D620
D619a: Type of antihypertensive	1	0	
D619a1: Hydralazine	1	0	
D619a2: Nifedipine	1	0	
D619a3: Labetalol			
D619a4: Methyldopa			
D619b: Dose of antihypertensive (mg) :	________________		
D620: *PLEASE WRITE A BRIEF SUMMARY OF THE CASE AND THEN DESCRIBE THE CONDITION OF THE WOMAN AT THE END OF OBSERVATION*			
D621: *PLEASE COMMENT ON THE QUALITY OF CARE PROVIDED:* *Was the woman left alone at any point even if there was a danger of convulsions? Was she treated respectfully? Informed of procedures? Was the situation chaotic or calm? Were there any major delays in needed treatment? If so, for what drugs/procedures and why? Were multiple health workers involved? Who?*			
**END SECTION 6**			

AFGHANISTAN MNH QUALITY OF CARE FACILITY ASSESSMENT
Tool D2: Labor and Delivery Observation Complication Checklist: PPH

Health facility visited (name):						
Health facility code (from HMIS and/or facility listing):						
Health worker code (assigned during listing):						

TYPE OF HEALTH FACILITY	
Specialized hospital .... [1]<br>Regional hospital .... [2]<br>Provincial hospital .... [3]<br>District hospital .... [4]<br>Comprehensive health center .... [5]<br>Basic health center .... [6]<br>Sub health center .... [7]	Private hospital ....[8]<br>Private clinic ....[9]<br>Other (specify) ....[10]<br>____
Province Name:	
District Name:	
City / Village Name:	
Name of Observer:	
Name of Team Leader	
Date of Visit: (dd/mm/yy)	__ __/ __ __/ __ __ __ __
Time of Visit: (hh:mm/am-pm)	__ __ :__ __/ __ __
Signature of Team Leader:	

D001: Facility name	D002: Facility number
D003: Observer number	D004: Today's date (day/month/year)

D700: Time observation began (time of diagnosis of PPH)	☐☐☐☐

Question	YES	NO	DK	Go to
D701: Time of birth	☐☐☐☐			
D702: Type of delivery:	**Code**			
Normal Vaginal	1			
Assisted Vaginal	2			
Cesarean	3			
D703: Is the woman: (*SELECT ALL THAT APPLY*)	**Code**			
Unconscious	1			
In shock	2			
D704: Does the woman have an atonic uterus?	1	0	98	
D705: Does the woman have a laceration?	1	0	98	
D706: Does the woman have retained placenta?	1	0	98	
D707: Type of provider(s) giving care: (*SELECT ALL THAT APPLY*)			98	
D707a. Midwife or community midwife	1	0	98	
D707b. Nurse	1	0	98	
D707c. General physician/clinician	1	0	98	
D707d. Obstetrician	1	0	98	
D707e. Pediatrician	1	0	98	
D707f. Other specialist	1	0	98	
D707g: Community health supervisor	1	0	98	
D707h: Community health worker	1	0	98	
D707i. Student (Nurse, Midwife, Medical)	1	0	98	
D708: Was the placenta already delivered?	1	0	98	
D709: Did you observe the use of AMTSL?	1	0		No→D710
D709a: Uterotonic given	1	0		No→D710b
	**Code**			
D709a1: Type of Uterotonic Given — Oxytocin	1			
Ergometrine	2			
Misoprostol	3			
D709a2: Time	☐☐☐☐			
D710a3: Dose:	________________			
D709b: Controlled cord traction	1	0	98	
D710: Time of delivery of placenta: (*observer: use 24h clock*)	☐☐☐☐ *Enter 9999 if placenta not delivered*			
D711: Is there a family member with the woman?	1	0		

MANAGEMENT/TREATMENT				
D712: Not including AMTSL, were any other Uterotonic given? (*IF MULTIPLE DRUGS WERE GIVEN, LIST FIRST DRUG AT D712a, SECOND DRUG AT D712b, AND THIRD DRUG AT 712c*)				
D712a: Does drug 1 given?	1	0		No→D713
D712a1: Drug 1:	___________________________________			
D712a2: Dose of Drug 1:	___________________________________			
D712a3: Route of Drug 1:	**Code**			
IV	1			
IM	2			
Oral	3			
Rectal	4			
Sublingual	5			
D712a4: Time of Drug 1:	[ ][ ][ ][ ]			
D712b: Does drug 2 given?	1	0		No --------→ D713
D712b1: Drug 2:	_________________________			
D712b2: Dose of Drug 2:	___________________________________			
D712b3: Route of Drug 2:	**Code**			
IV	1			
IM	2			
Oral	3			
Rectal	4			
Sublingual	5			
D712e4: Time of Drug 2: (*observer: use 24h clock*)	[ ][ ][ ][ ]			
D712c: Does drug 3 given?	1	0		No--→ D713
D712c1: Drug 3: (*Specify*):	___________________________________			
D712c2: Dose of Drug 3:	___________________________________			
D712c3: Route of Drug 3:	**Code**			
IV	1			
IM	2			
Oral	3			
Rectal	4			
Sublingual	5			
D712i4: Time of Drug 3: (*observer: use 24h clock*)	[ ][ ][ ][ ]			
D713: Was uterine massage performed?	1	0		
D714: Was an IV line inserted?	1	0		
D715: Were vital signs checked at least once?	1	0	98	
D716: Did the woman receive blood?	1	0		No→D717
D716a: Total Number of Units of Blood				
D716b: Time: (*observer: use 24h clock*)	[ ][ ][ ][ ]			
D717: Did the woman have: (*SELECT ALL THAT APPLY*)				
D717a: Removal of pieces of placenta	1	0	98	
D717b: Manual removal of placenta	1	0	98	
D717c: Uterine Exploration	1	0	98	
D717d: Uterine bimanual compression	1	0	98	
D717e: Repair of vaginal/cervical laceration	1	0	98	
D717f: Uterine balloon tamponade	1	0	98	
D717g: Aortic compression	1	0	98	
D717h: Other (*Specify*) __________________	1	0	09	
D718: From the records, what was the estimated blood loss?	[ ][ ][ ][ ]			
D719: From the records, was it recorded as PPH in the delivery	1	0	98	

register?	
D720: *PLEASE WRITE A BRIEF SUMMARY OF THE CASE AND THEN DESCRIBE THE CONDITION OF THE WOMAN AT THE END OF OBSERVATION*	
D721: *PLEASE COMMENT ON THE QUALITY OF CARE PROVIDED:*<br>*Were the steps followed in an orderly fashion (treat for atony; look for tears; consider incomplete expulsion of placenta; treat shock)? Was the woman left alone? Was she treated respectfully? Informed of procedures? Was the situation chaotic or calm? Were there any major delays in needed treatment? If so, for what drugs/procedures and why? Were multiple health workers involved? Who?*	
**End of Section 7**	

AFGHANISTAN MNH QUALITY OF CARE FACILITY ASSESSMENT

Tool D3: Labor and Delivery Observation Complication Checklist: Newborn Resuscitation

Health facility visited (name):						
Health facility code<br>(from HMIS and/or facility listing):						
Health worker code (assigned during listing):						

TYPE OF HEALTH FACILITY	
Specialized hospital .... [1]<br>Regional hospital .... [2]<br>Provincial hospital .... [3]<br>District hospital .... [4]<br>Comprehensive health center .... [5]<br>Basic health center .... [6]<br>Sub health center .... [7]	Private hospital ....[8]<br>Private clinic ....[9]<br>Other (specify) ....[10]<br>____
Province Name:	
District Name:	
City / Village Name:	
Name of Observer:	
Name of Team Leader	
Date of Visit: (dd/mm/yy)	__ __/ __ __/ __ __ __ __
Time of Visit: (hh:mm/am-pm)	__ __ :__ __/ __ __
Signature of Team Leader:	

AFGHANISTAN MNH QUALITY OF CARE FACILITY ASSESSMENT

Tool D3: Labor and Delivery Observation Complication Checklist: Newborn Resuscitation

D001: Facility name	D002: Facility number
D003: Observer number	D004: Today's date (day/month/year)

SECTION 8: NEWBORN RESUSCITATION	
D800: Record time resuscitation started (*Observer:* Please use 24 hr clock)	☐☐☐☐
D801: Location of resuscitation	**Code**
Table or space in delivery room	1
Outside of delivery room	2

RECORD WHETHER THE PROVIDER CARRIED OUT THE FOLLOWING STEPS AND/OR EXAMINATIONS: (SOME OF THE FOLLOWING STEPS MAY BE PERFORMED SIMULTANEOUSLY OR BY MORE THAN ONE PROVIDER)

**Question**	**Yes**	**No**	**DK**	**Go to**
D802: Clears the airway by suctioning the mouth first and then the nose	1	0	98	
D803: Stimulates baby with back rubbing while drying the baby	1	0	98	
D804: *OBSERVER:* Does newborn start to breathe or cry spontaneously?	1	0		Yes→D821
D805: Ties or clamps cord immediately	1	0	98	
D806: Cuts cord with sterile surgical blade or sterile scissors	1	0	98	
D807: Places the newborn on his/her back with the head in a slightly extended position to open the airway	1	0	98	
D808: Tells the woman (and her support person) what is going to be done, providing support and reassurance	1	0	98	
D809: Checks mouth, back of throat and nose for secretions, and clears if necessary	1	0	98	
D810: Places the correct-sized mask on the newborn's face so that it covers the chin, mouth and nose (but not eyes)	1	0	98	
D811: Checks the seal by ventilating two times and observing the rise of the chest	1	0	98	
D812: Record time 1st ventilation attempt started	☐☐☐☐			
D813: *OBSERVER:* Is newborn's chest rising in response to ventilation?	1	0		Yes→D825
D813a: Calls for help	1	0	98	

IF NEWBORN'S CHEST IS NOT RISING AFTER TWO ATTEMPTS TO READJUST, OBSERVER SHOULD CALL FOR SUPERVISOR TO INTERVENE. IF A HEALTH WORKER COMPETENT IN RESUSCITATION IS NOT AVAILABLE, OBSERVER MAY CHOOSE TO INTERVENE.

**Question**	**Yes**	**No**	**DK**	**Go to**
D814: Ventilates at a rate of 30-50 breaths/minute	1	0	98	
D815: Condition of newborn at assessment	**Code**			
Heart rate is greater than100 beats per minute	1			→D820
Heart rate is less than100 beats per minute	2			
D816: Continues ventilation and baby cries before 10 minutes	1	0		
D817: Conducts assessment of newborn breathing after prolonged ventilation (10 minutes)	1	0		No→D819
D818: Condition of newborn at assessment	**Code**			
Breathing well (Respiration rate at or near 40breaths/minute and no chest indrawing)	1			→D820
Not breathing well	2			
No spontaneous breathing	3			
D819: Continues ventilation	1	0		
D820: Record time that resuscitation actions ended (or time of death if baby died)	☐☐☐☐			

Question	Yes	No	DK	Go to
D821: OBSERVER: Is the baby alive and breathing spontaneously? (Select No if newborn died)	1	0		No→D823
D821a: Is baby placed on mother's abdomen "skin to skin"?	1	0		
D822: Arranges transfer to special care either in facility or to outside facility	1	0	98	
D823: Explains to the mother (and her support person if available) what happened	1	0	98	
D824: Listens to mother and responds attentively to her questions and concerns	1	0	98	
**CLEAN UP AFTER NEWBORN RESUSCITATION**				
D825: Wipes bag and mask with 0.5% chlorine solution	1	0	98	
D826: Decontaminates reusable suction devices, washes and leaves to dry	1	0	98	
D827: Washes his/her hands with soap and water or uses alcohol hand rub	1	0	98	
**QUESTIONS FOR OBSERVER**				
D828: OBSERVER: Did you call for help or intervene during the resuscitation to save the life of newborn?	1	0		
D829: *PLEASE COMMENT ON THE QUALITY OF CARE PROVIDED: Was mother treated respectfully? Informed of procedures to her baby? Was the situation chaotic or calm? Were there any major delays in needed treatment? It is important to capture delays in helping the baby to breathe. If so, for what drugs/procedures and why? Were multiple health workers involved? Who were the health worker who? If newborn did not survive, describe the circumstances. Was the mother counseled about the death of newborn?*				
END OF SECTION 8 – Return to D403				

AFGHANISTAN MNH QUALITY OF CARE FACILITY ASSESSMENT
Tool E: PNC Observation Checklist

Health facility visited (name):						
Health facility code (from HMIS and/or facility listing):						
Health worker code (assigned during listing):						

TYPE OF HEALTH FACILITY	
Specialized hospital .... [1]<br>Regional hospital .... [2]<br>Provincial hospital .... [3]<br>District hospital .... [4]<br>Comprehensive health center .... [5]<br>Basic health center .... [6]<br>Sub health center .... [7]	Private hospital ....[8]<br>Private clinic ....[9]<br>Other (specify) ....[10]<br>____
Province Name:	
District Name:	
City / Village Name:	
Name of Observer:	
Name of Team Leader	
Date of Visit: (dd/mm/yy)	__ __/ __ __/ __ __ __ __
Time of Visit: (hh:mm/am-pm)	__ __ :__ __/ __ __
Signature of Team Leader:	

E001: Facility name	E002: Facility number
E003: Observer number	E004: Today's date (day/month/year)

BEFORE OBSERVING THE CONSULTATION, OBTAIN PERMISSION FROM BOTH THE SERVICE PROVIDER AND THE CLIENT. MAKE SURE THAT THE PROVIDER KNOWS THAT YOU ARE NOT THERE TO EVALUATE HIM OR HER, AND THAT YOU ARE NOT AN "EXPERT" TO BE CONSULTED DURING THE SESSION.

Hello, I am _________________. I am a [midwife / physician] representing the Ministry of Public Health and the USAID-funded HEMAYAT Project. We are conducting a study of health facilities in this country, with the goal of finding ways to improve maternal and newborn health services. We are recruiting health care providers in more than 200 facilities across the country to participate in this study. Specifically, we are recruiting those who are providers of maternal and newborn health services. May I continue?

READ TO HEALTHCARE WORKER.
I would like to be present to observe the duration of your consultation with this client in order to understand how postnatal care services are provided in this facility.

There will be no direct benefit to you from being in this study. Information from this observation is confidential. Neither your name nor that of the client will be recorded. The information acquired during this observation may be used by the Ministry of Public Health or other organizations to improve services, or for research on health services; however, neither your name nor the name of your clients will be entered into the database. You do not have to agree to be in this study, and you may change your mind at any time.

Do you have any questions for me? You may call the HEMAYAT Project Technical Director, Dr. Partamin, at 0700020686. You may also contact the Ministry of Public Health Ethics Committee which approved this study by calling Dr. Sayed Murtaza Hofiani at 070055560 with any problems or concerns about the study.

Do you have any questions for me? If at any point you feel uncomfortable you can ask me to leave. However, we hope you will not mind our observing your consultation.

E005: *ASK HEALTH WORKER:* Do I have your permission to be present at this consultation?
☐ Yes, consent is given → go to E006
☐ No, consent is not given → assessment of this healthcare worker must END.

Interviewer's Signature and date: (indicates respondent's willingness to participate): ____________________

E006: Health worker line number (from staff listing):	E007: Sex of health worker<br>Male 0<br>Female 1
E008: Health worker category	**CODE**
Midwife or community midwife	1
Nurse or community health nurse	2
General physician/clinician	3
Obstetrician	4
Pediatrician	5
Other specialist	6
Community health supervisor	7
Community health workers	8
Student (nurse, midwife, medical)	9

READ ORAL CONSENT SCRIPT TO CLIENT.

Good day. , I am ___________________. I am a [midwife / physician] representing the Ministry of Public Health and the USAID-funded HEMAYAT Project. We are conducting a study of health facilities to improve maternal and newborn health services. I would like to be present to observe your postnatal care consultation in order to better understand how health services are provided in this facility. We expect your postnatal care consultation will last less than one hour.

Other patients in this facility are also being asked to participate. Please know that whether you decide to allow me to observe your visit is completely voluntary, and you may quit at any time. Whether you agree to participate or not will not affect the services you receive. While there are no direct benefits to you from being in this study, we expect your participation will help improve maternity health services in Afghanistan. We are not evaluating your postnatal care provider or this facility. Neither your name nor the date of services will be provided on any shared data, so your identity and any information about you will remain completely confidential. You do not have to agree to be in this study, and you may change your mind at any time.

Do you have any questions for me? You may call the HEMAYAT Project Technical Director, Dr. Partamin, at 0700020686. You may also contact the Ministry of Public Health Ethics Committee which approved this study by calling Dr. Sayed Murtaza Hofiani at 070055560 with any problems or concerns about the study.

If at any point you feel uncomfortable you can ask me to leave. However, we hope you won't mind our observing your consultation. Do you have any questions for me?

E009: *ASK CLIENT:* Do I have your permission to be present while you are receiving services today?
☐ Yes, consent is given → go to E010
☐ No, consent is not given → observation of this client must <u>END</u>; if available, approach another client for participation.

Interviewer's Signature and date: (indicates respondent's willingness to participate): ____________________

E010: Client Code

SECTION 1: PRE-DISCHARGE OBSERVATION (INPATIENT ROUNDS)				
E100: Record time observation started	☐☐☐☐			
**Question**	**Yes**	**No**	**DK**	**Go to**
E101: Did the health worker greet the mother (and others present) in a friendly and respectful manner?	1	0	98	
E102: Did the health worker introduce herself and title (midwife, nurse, etc)?	1	0	98	
E103: Did the health worker ask about the location of delivery?	1	0	98	No / DK → E104
E103a: Place of delivery	**CODE**			
Home	1			
Health Facility	2			
Other	95	Specify: ___________________		

RECORD WHETHER THE PROVIDER CARRIED OUT THE FOLLOWING STEPS AND / OR EXAMINATIONS: (SOME OF THE FOLLOWING STEPS MAY BE PERFORMED SIMULTANEOUSLY OR BY ANOTHER PROVIDER

INITIAL ASSESSMENT AND EXAMINATION OF MOTHER				
**Question**	**Yes**	**No**	**DK/NA**	**Go to**
E104:: Checks for danger signs in the mother:				
E104a: Convulsions / loss of consciousness	1	0	98	
E104b: Difficulty breathing	1	0	98	
E104c: Excessive vaginal bleeding	1	0	98	
E104d: Fever or chills	1	0	98	
E104e: Visual disturbance	1	0	98	
E104f: Severe headache	1	0	98	
E104g: Other please specify:	1	0	95	
E105: Conducts any of the following procedures for the mother:				
E105a: Takes pulse	1	0	98	
E105b: Takes blood pressure	1	0	98	
E105c: Takes temperature	1	0	98	
E105d: Examines abdomen	1	0	98	
E105e: Checks fundus and massages if soft	1	0	98	
E105f: Checks perineum / episiotomy if perineal trauma during delivery	1	0	98	
E105g: Checks condition of mother's cesarean scar	1	0	98	
E105h: Other please specify:	1	0	95	
E106: Asks the mother if she is breastfeeding	1	0	98	No / DK → E107
E106a: OBSERVER: Is the mother or baby having any problems breastfeeding?	1	0	98	No / DK → E107
E106a1: Assesses breastfeeding techniques	1	0	98	
E107: Counsels the mother on any of the following maternal danger signs:				
E107a: Fever	1	0	98	
E107b: Foul smelling, green, or excessive vaginal discharge	1	0	98	
E107c: Difficulty breathing	1	0	98	
E107d: Heavy vaginal bleeding	1	0	98	
E107e: Severe lower abdominal pains	1	0	98	
E107f: Severe headaches / blurred vision	1	0	98	
E107g: Dizziness / loss of consciousness	1	0	98	
E107h: Convulsions	1	0	98	
E107j: Incontinence of urine or stool	1	0	98	
E107k: Low mood (feelings of anxiety, hallucinations, sadness, etc)	1	0	98	
E107l: Other please specify:	1	0	95	
E108: OBSERVER: Did the health worker diagnose a complication?	1	0	98	No / DK → E109
E108a: If yes, what was the diagnosis:				
E108a1: Systemic bacterial infection/ sepsis	1	0	98	
E108a2: Local bacterial infection (in perineum area or cesarean scar)	1	0	98	

E108a3: Hypertension	1	0	98	
E108a4: Malaria	1	0	98	
E108a5: Severe anemia	1	0	98	
E108a6: Secondary PPH	1	0	98	
E108a7: Don't know	1	0	98	
E108a8: Other (Specify): _________________________________	1	0	95	
**INITIAL ASSESSMENT AND EXAMINATION OF NEWBORN**				
**Question**	**Yes**	**No**	**DK**	**Go to**
E109: Discusses the baby's birth weight with the mother	1	0	98	
E110: OBSERVER: Was the baby born before 37 weeks or is the baby's weight < 2500gms?	1	0	98	
E111: Asks if the baby has any of the following danger signs:	1	0		NO → E113
E111a: Not able to feed	1	0	98	
E111b: Convulsions	1	0	98	
E111c: Fast breathing (breathing rate of 60 breaths per minute or higher)	1	0	98	
E111d: Severe chest in-drawing (stridor)	1	0	98	
E111e: No spontaneous movement	1	0	98	
E111f: Fever (temperature of 37.5 degrees C or higher)	1	0	98	
E111g: Low body temperature (temperature less than 36.5 degrees C)	1	0	98	
E111h: Any yellow coloring in the first 24 hours of life or yellow palms and soles at any age	1	0	98	
E112: Conducts any of the following procedures for the baby:				
E112a: Takes baby's axillary temperature	1	0	98	
E112b: Checks and counts baby's breathing	1	0	98	
E112c: Checks baby's eyes	1	0	98	
E112d: Checks baby's mouth	1	0	98	
E112e: Checks cord	1	0	98	
E112f: Checks baby passing stool	1	0	98	
E112h: Other please specify:	1	0	95	

RECORD WHETHER THE PROVIDER CARRIED OUT THE FOLLOWING STEPS AND / OR EXAMINATIONS:

**INFECTION PEVENTION PRACTICES OF PROVIDER**				
**Question**	**Yes**	**No**	**DK**	**Go to**
E113: Conducted any of the following:				
E113a: Washed hands with soap or used hand sanitizer before examining the baby	1	0	98	
E113b: Washed hands with soap or used hand sanitizer before examining the mother	1	0	98	
E113c: Washed hands with soap or used hand sanitizer after examining the baby	1	0	98	
E113d: Washed hands with soap or used hand sanitizer after examining the mother	1	0	98	
**COUNSELING**				
**Question**	**Yes**	**No**	**DK**	**Go to**
E114: Counseled the mother on any of the following:				
E114a: Importance of exclusive breastfeeding for 6 months (explaining this means not even giving water/other fluids to the baby)	1	0	98	
E114b: Keeping the baby warm	1	0	98	
E114c: Cord care, including application of chlorhexidine	1	0	98	
E114d: Vaccination schedule, location and importance	1	0	98	
E114e: PNC follow up at day 3, day 14, week 6	1	0	98	
E114f: Maternal and newborn signs that should trigger immediate care seeking	1	0	98	
E114g: Hand washing and general hygiene	1	0	98	
E114h: Other please specify:	1	0	95	
E115: Counseled the mother on any of the following for healthy timing and spacing of pregnancy?				

E115a: Correct use of lactational amenorrhea as a temporary method? *(Correct use of LAM requires exclusive or near exclusive breastfeeding while baby is less than six months and menses has not yet returned)*	1	0	98	
E115b: Health benefits of longer interpregnancy intervals (or risks of intervals less than 2 years) (2 year interim)	1	0	98	
E115c: Fertility return / risk of pregnancy if not exclusively breastfeeding or baby is 6 months of age or older	1	0	98	
E115d: Other please specify:	1	0	95	
E116: Asked the mother about her intention for future pregnancy (spacing or limiting)?	1	0	98	NO → E117
E116a: If she has already chosen a family planning method?	1	0	98	
E116b: If she has not tolerated a family planning method in the past?	1	0	98	
E117: Discussed options available for spacing, if appropriate?(Select all mentioned):	1	0	98	NO → E118
E117a: Lactational amenorrhea				
E117b: PPIUCD and interval IUCD	1	0	98	
E117c: Implants	1	0	98	
E117d: Injectable	1	0	98	
E117e: Progestin – only pills (POP)	1	0	98	
E117f: Combined oral contraceptives	1	0	98	
E117g: Condoms	1	0	98	
E118: Discussed permanent family planning options available, if appropriate?(Select all mentioned):	1	0	98	NO → E119
E118a: Vasectomy	1	0	98	
E118b: Bilateral tubal ligation	1	0	98	
E119: Discussed when to return for family planning method (if not provided at visit)	1	0	98	
E120: Did the health worker document PPFP counseling and method chosen in a register?	1	0	98	

**PROVIDER ATTITUDES**			
E121: Did the health worker do any of the following:			
E121a: Explain to the mother what was going to be done prior to examination	1	0	98
E121b: Ask whether the mother had any questions and / or encouraged questions	1	0	98
E121c: Show concern and respect to client culture, beliefs, and ideas	1	0	98
E121d: Speak using easy-to-understand language for the client	1	0	98
E121e: Use any visual aids for health education or counseling during the consultation	1	0	98
E121f: Record / revised information on a patient card or register	1	0	98
E121g: Refer the mother to a family planning provider to receive her method of choice	1	0	98

**OUTCOMES**	
E122: Record outcome of visit for baby:	**Code**
Baby goes home	1
Baby referred to lab or other provider at same facility	2
Baby admitted to same facility	3
Baby referred to other facility	4
Baby started first dose of antibiotic and was referred	5
Baby started treatment and hospitalization in same facility	6
Don't know	98
E123: Record outcome of visit for mother:	
Mother goes home	1
Mother referred to lab or other provider at same facility	2
Mother admitted to same facility	3
Mother referred to other facility	4

Don't know	98
E124: Record the time the consultation ended	☐☐☐☐

E125: PLEASE COMMENT ON THE QUALITY OF CARE PROVIDED:<br>*Were the steps followed in an orderly fashion? Was the newborn / infant left alone? Was the mother treated respectfully? Was the caregiver informed of procedures? Was the situation chaotic or calm? Were there any major delays in needed treatment? If so, for what drugs / procedures and why? Were multiple health workers involved? Who? Was baby kept warm during examination and procedures?*
**END OF SECTION 1. END OF OBSERVATION.**
